# Supplementary material for: Accurate integration of single-cell DNA and RNA for analyzing intratumor heterogeneity using MaCroDNA
Source: Nat Commun. 2023 Dec 13;14:8262. doi: 10.1038/s41467-023-44014-3 (PMC10719311; doi:10.1038/s41467-023-44014-3)
Supplement: Supplementary file 1 — Supplementary Information file [file 41467_2023_44014_MOESM1_ESM.pdf]

Supplementary material for

Accurate integration of single-cell DNA and RNA for analyzing intratumor heterogeneity using MaCroDNA

Mohammadamin Edrisi[1,*] Xiru Huang[1] Huw A. Ogilvie[1,*]
Luay Nakhleh[1,*]
[1]Department of Computer Science, Rice University, Houston, Texas, USA.
[*]Corresponding author

1 Supplementary Figures and Tables

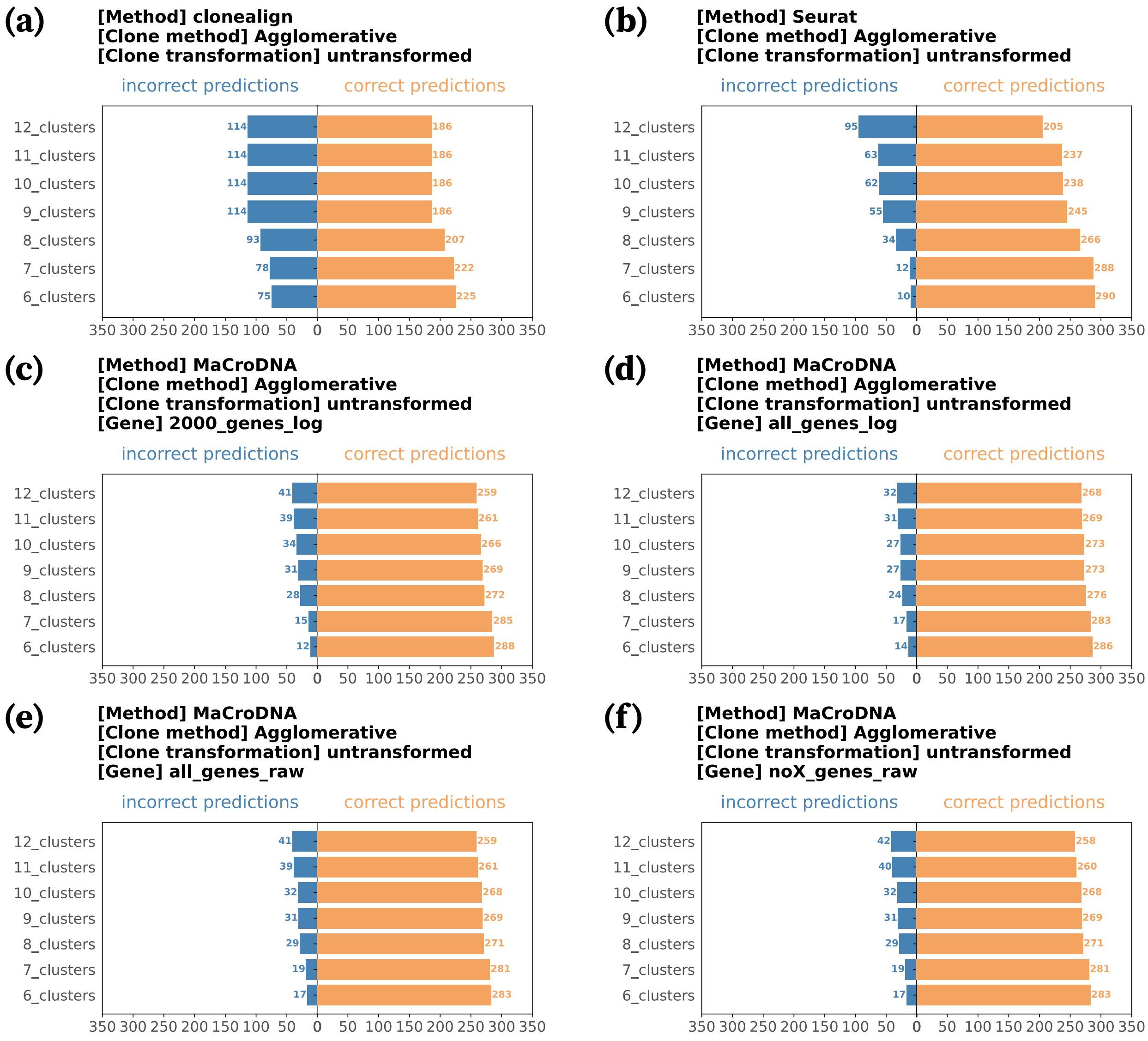

Figure S1: **Results of agglomerative clustering method, using untransformed data for clustering.** Each panel is a mirror plot showing the accuracy of a method on the three CRC patients under different clustering resolutions: **(a)** for clonealign, **(b)** for Seurat, and **(c-f)** for MaCroDNA with different preprocessing procedures on its input data. In panel **(a)**, the input of clonealign is the original data without the genes on X-chromosome. In panel **(b)**, the input of Seurat is the log-transformed data with the top 2000 genes selected. The input of MaCroDNA has four different settings: in **(c)**, `2000_genes_log` is the same as the input of Seurat, in **(d)**, `all_genes_log` uses the same log-transformation as Seurat but all genes are kept, in **(e)**, `all_genes_raw` is the original data with all genes, and in **(f)**, `noX_genes_raw` is the same as the input of clonealign: the original values without the genes on X-chromosome. In each panel, we performed the corresponding method on each patient separately. Source data are provided as a Source Data file.

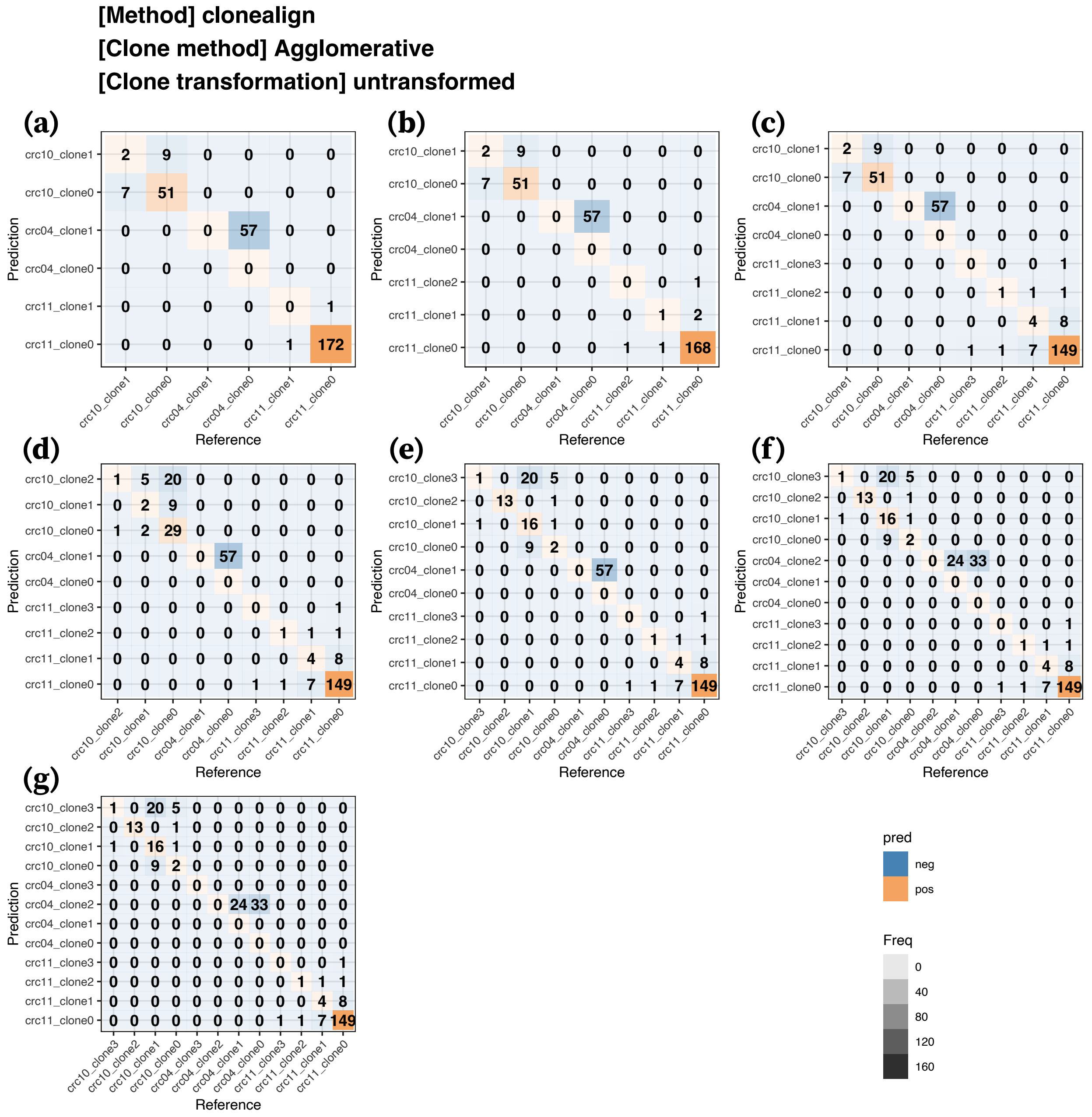

Figure S2: **clonealign results using agglomerative clustering method and the original data for clustering.** Each panel shows the accuracy of clonealign on the three CRC patients in the form of a confusion matrix plot under the clustering resolution of six **(a)**, seven **(b)**, eight **(c)**, nine **(d)**, 10 **(e)**, 11 **(f)**, and 12 **(g)** clusters. The input to clonealign is the original data without the genes on X-chromosome. The data for clustering is the original untransformed data. In each panel, we performed clonealign on each patient separately. Source data are provided as a Source Data file.

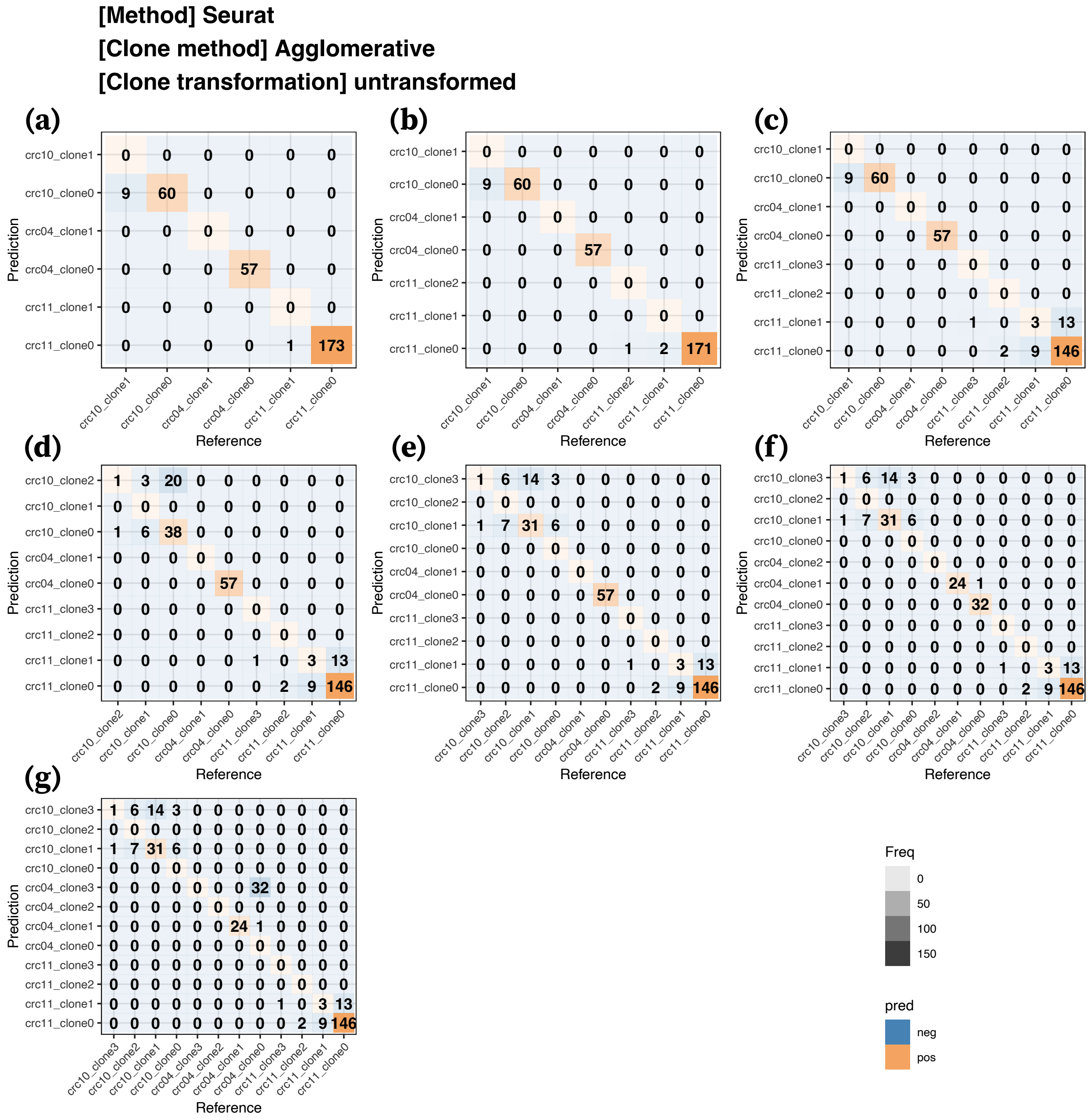

Figure S3: **Seurat results using agglomerative clustering method and the original data for clustering.** Each panel shows the accuracy of clonealign on the three CRC patients in the form of a confusion matrix plot under the clustering resolution of six **(a)**, seven **(b)**, eight **(c)**, nine **(d)**, 10 **(e)**, 11 **(f)**, and 12 **(g)** clusters. The input to Seurat is the log-transformed data with the top 2000 genes having been selected. The data for clustering is the original data. In each panel, we performed Seurat on each patient separately. Source data are provided as a Source Data file.

[Method] MaCroDNA
[Clone method] Agglomerative
[Clone transformation] untransformed
[Gene] 2000_genes_log

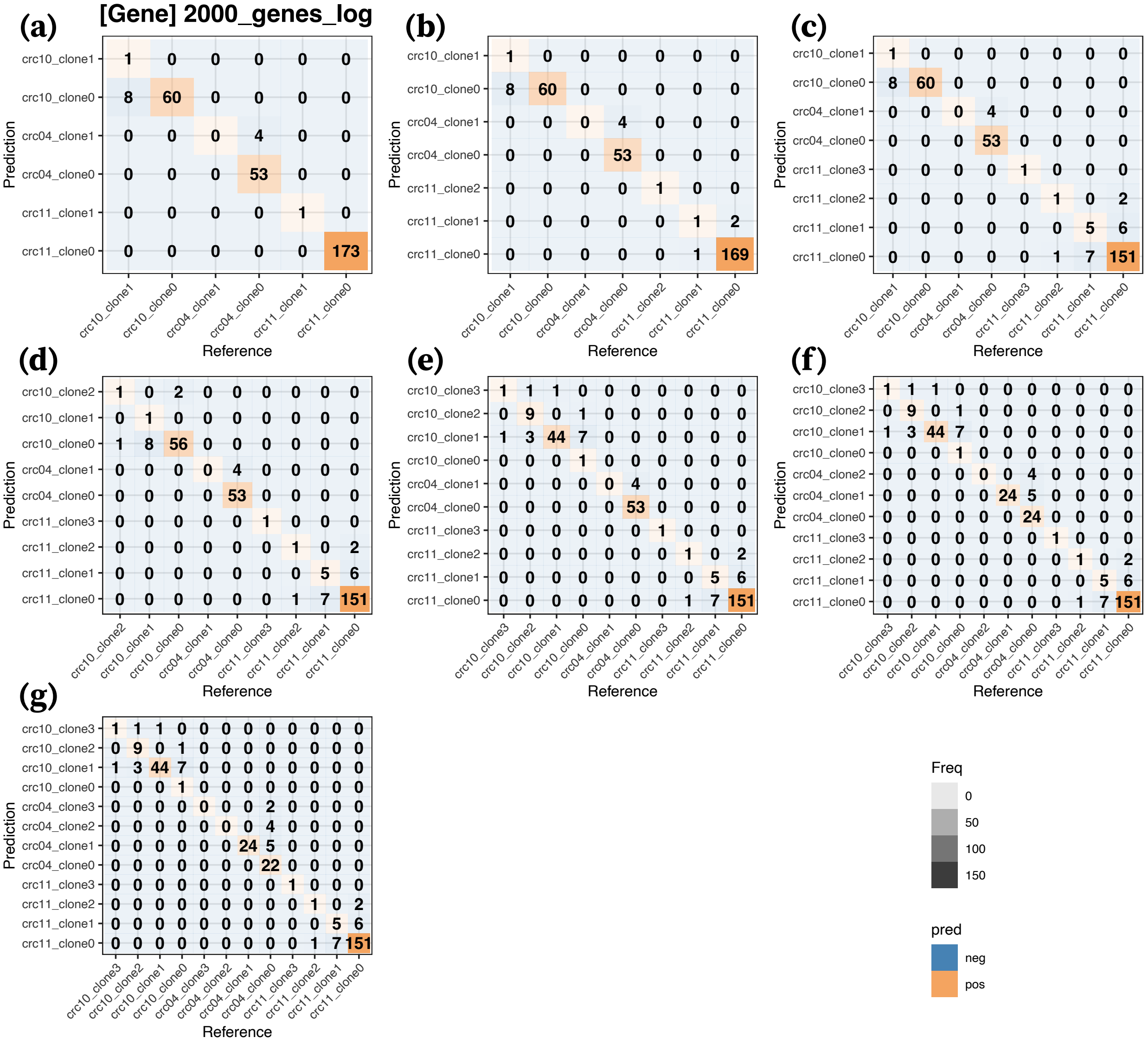

Figure S4: **MaCroDNA results using agglomerative clustering method and the original data for clustering.** Each panel shows the accuracy of clonealign on the three CRC patients in the form of a confusion matrix plot under the clustering resolution of six **(a)**, seven **(b)**, eight **(c)**, nine **(d)**, 10 **(e)**, 11 **(f)**, and 12 **(g)** clusters. The input to MaCroDNA is the same as the input for Seurat. The input is the log-transformed data with the top 2000 genes having been selected. The data for clustering is the original data. In each panel, we performed MaCroDNA on each patient separately. Source data are provided as a Source Data file.

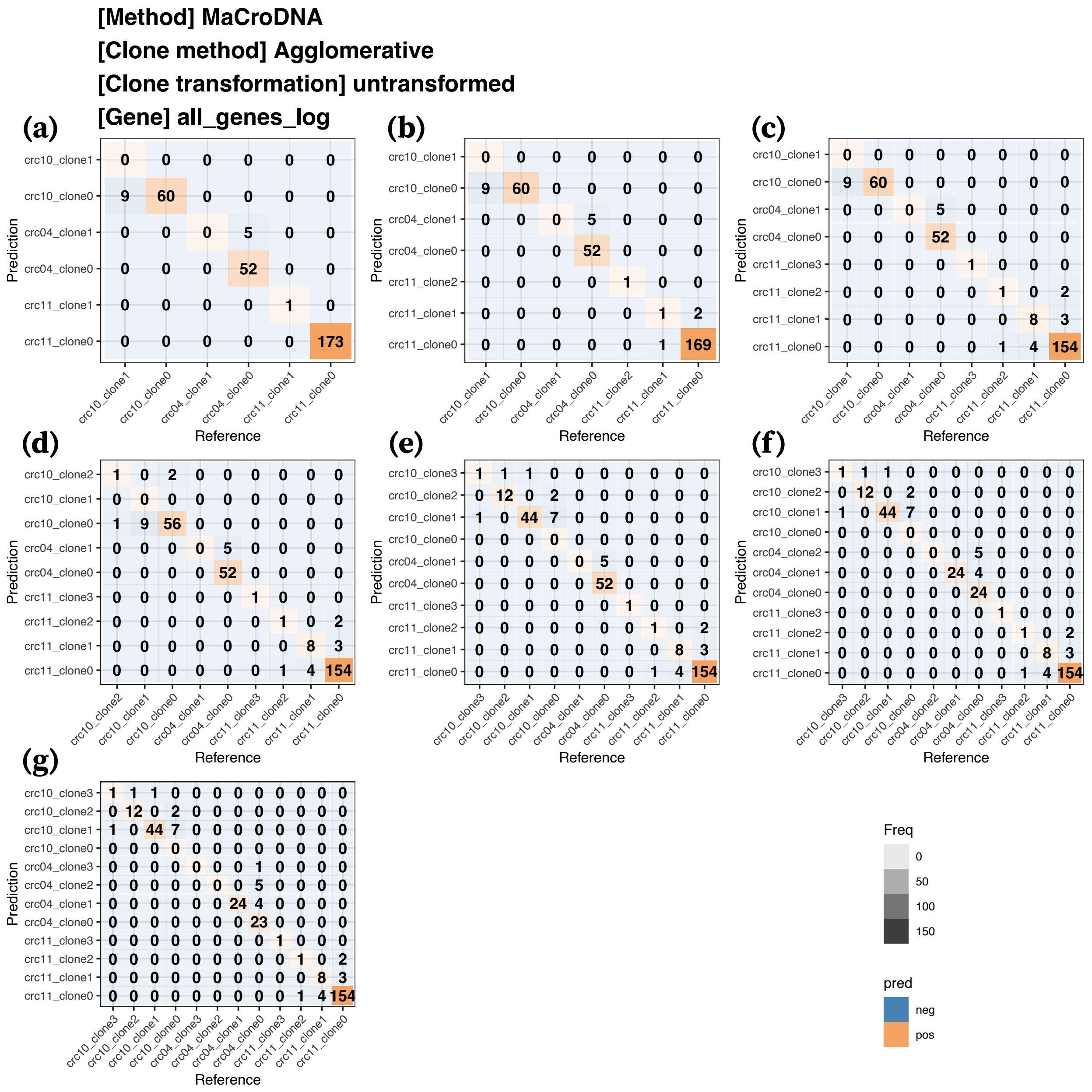

Figure S5: **MaCroDNA results using agglomerative clustering method and the original data for clustering.** Each panel shows the accuracy of clonealign on the three CRC patients in the form of a confusion matrix plot under the clustering resolution of six **(a)**, seven **(b)**, eight **(c)**, nine **(d)**, 10 **(e)**, 11 **(f)**, and 12 **(g)** clusters. The input to MaCroDNA is the log-transformed data, which is the same transformation applied to the input for Seurat, with the difference being that here, all genes are used. The data for clustering is the original data. In each panel, we performed MaCroDNA on each patient separately. Source data are provided as a Source Data file.

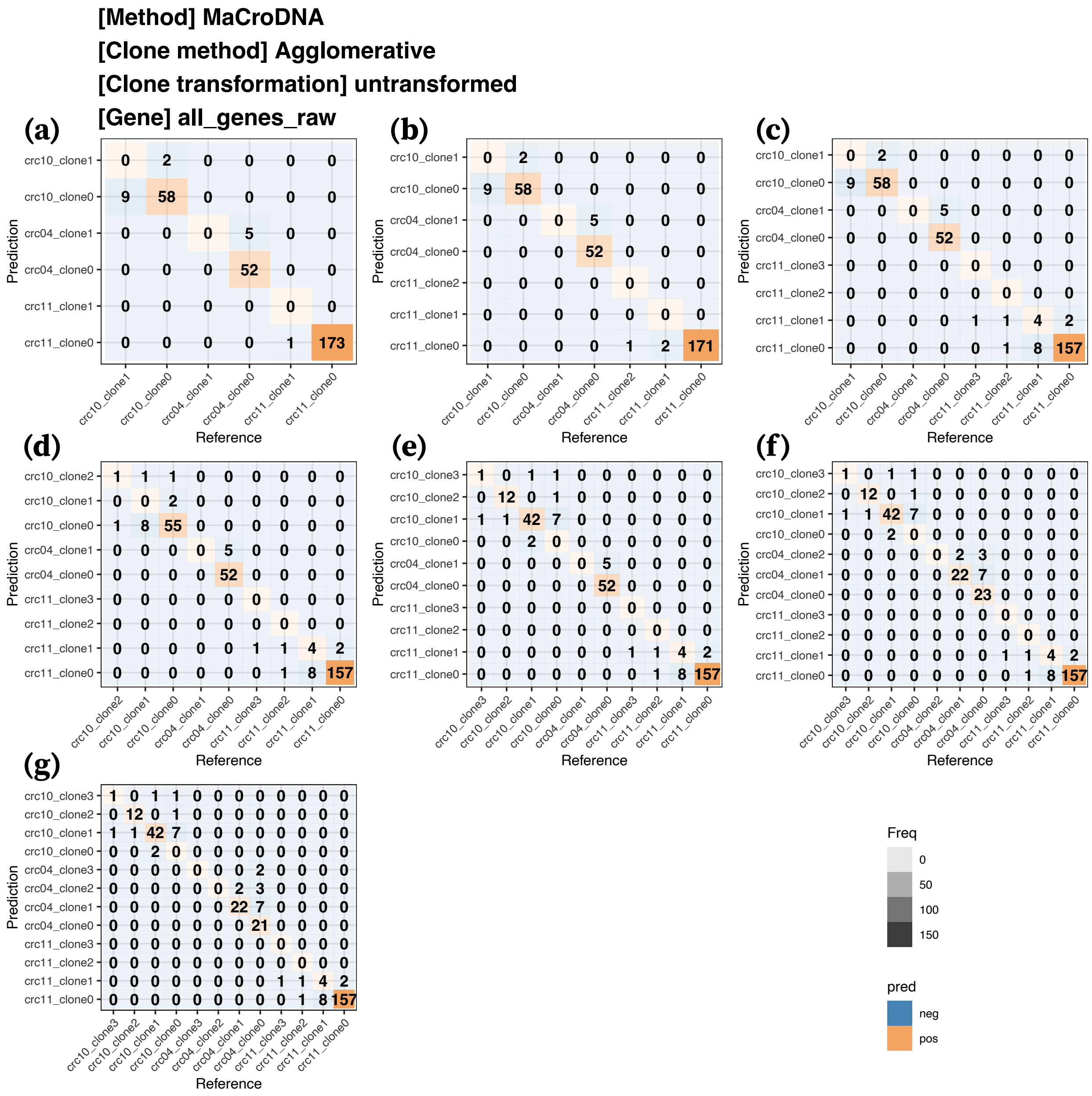

Figure S6: **MaCroDNA results using agglomerative clustering method and the original data for clustering.** Each panel shows the accuracy of clonealign on the three CRC patients in the form of a confusion matrix plot under the clustering resolution of six **(a)**, seven **(b)**, eight **(c)**, nine **(d)**, 10 **(e)**, 11 **(f)**, and 12 **(g)** clusters. The input to MaCroDNA is the original data with all the genes. The data for clustering is the original data. In each panel, we performed MaCroDNA on each patient separately. Source data are provided as a Source Data file.

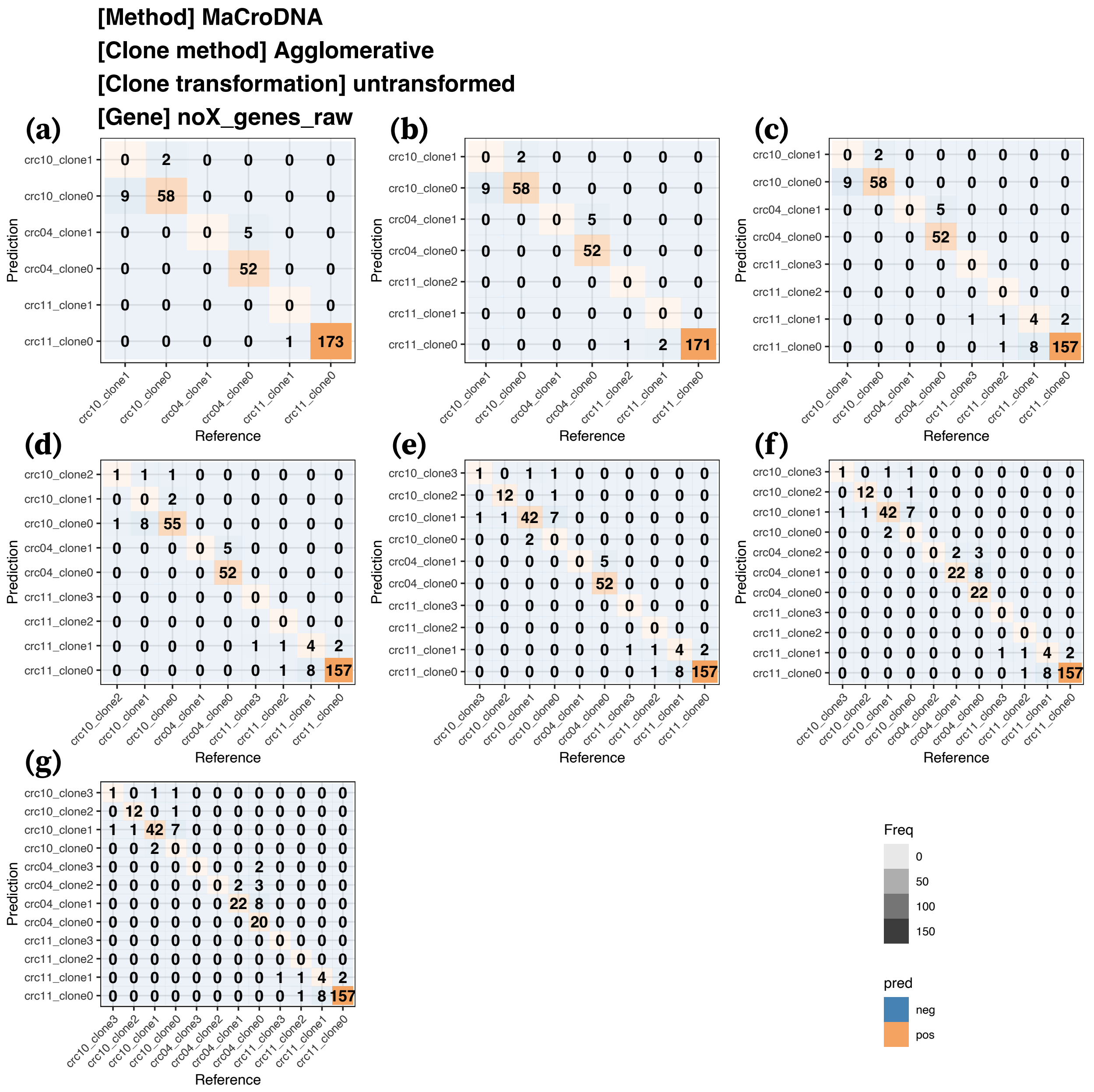

Figure S7: **MaCroDNA results using agglomerative clustering method and the original data for clustering.** Each panel shows the accuracy of clonealign on the three CRC patients in the form of a confusion matrix plot under the clustering resolution of six **(a)**, seven **(b)**, eight **(c)**, nine **(d)**, 10 **(e)**, 11 **(f)**, and 12 **(g)** clusters. The input to MaCroDNA is the original data without the genes on X-chromosome, the same as the input for clonealign. The data for clustering is the original data. For each panel, we performed MaCroDNA on each patient separately. Source data are provided as a Source Data file.

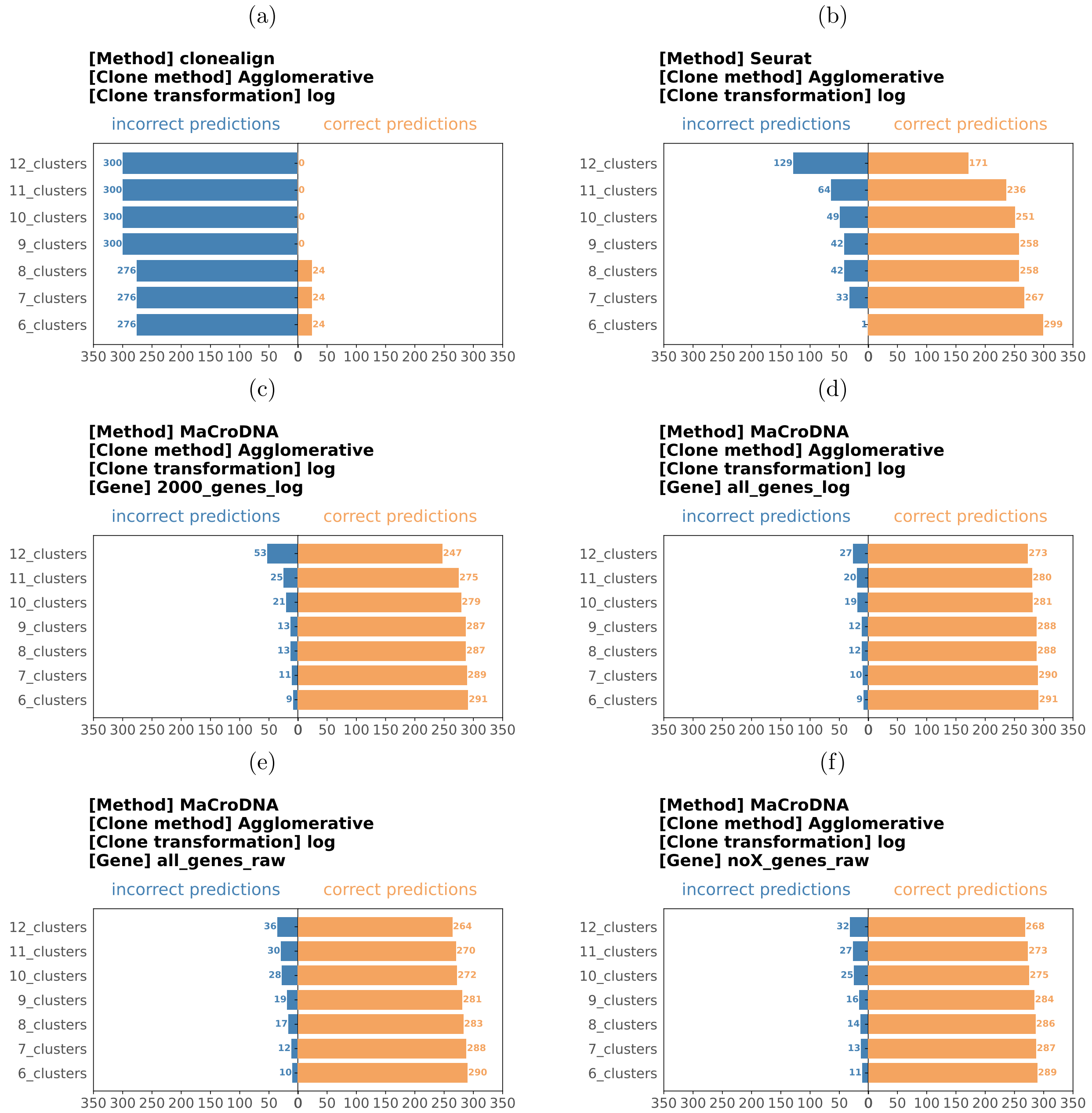

Figure S8: **Results of agglomerative clustering method, using log-transformed data for clustering.** Each panel is a mirror plot showing the accuracy of a method on the three CRC patients under different clustering resolutions: **(a)** for clonealign, **(b)** for Seurat, and **(c-f)** for MaCroDNA with different preprocessing procedures on its input data. In panel **(a)**, the input of clonealign is the original data without the genes on X-chromosome. In panel **(b)**, the input of Seurat is the log-transformed data with the top 2000 genes having been selected. The input of MaCroDNA has four different preprocessing settings: in **(c)**, `2000_genes_log` is the same as the input of Seurat, in **(d)**, `all_genes_log` uses the same log-transformation as Seurat but all genes are used, in **(e)**, `all_genes_raw` is the original data with all genes, and in **(f)**, `noX_genes_raw` is same as the input of clonealign: original values without the genes on X-chromosome. For each panel, we performed the corresponding method on each patient separately. Source data are provided as a Source Data file.

[Method] clonealign
[Clone method] Agglomerative
[Clone transformation] log

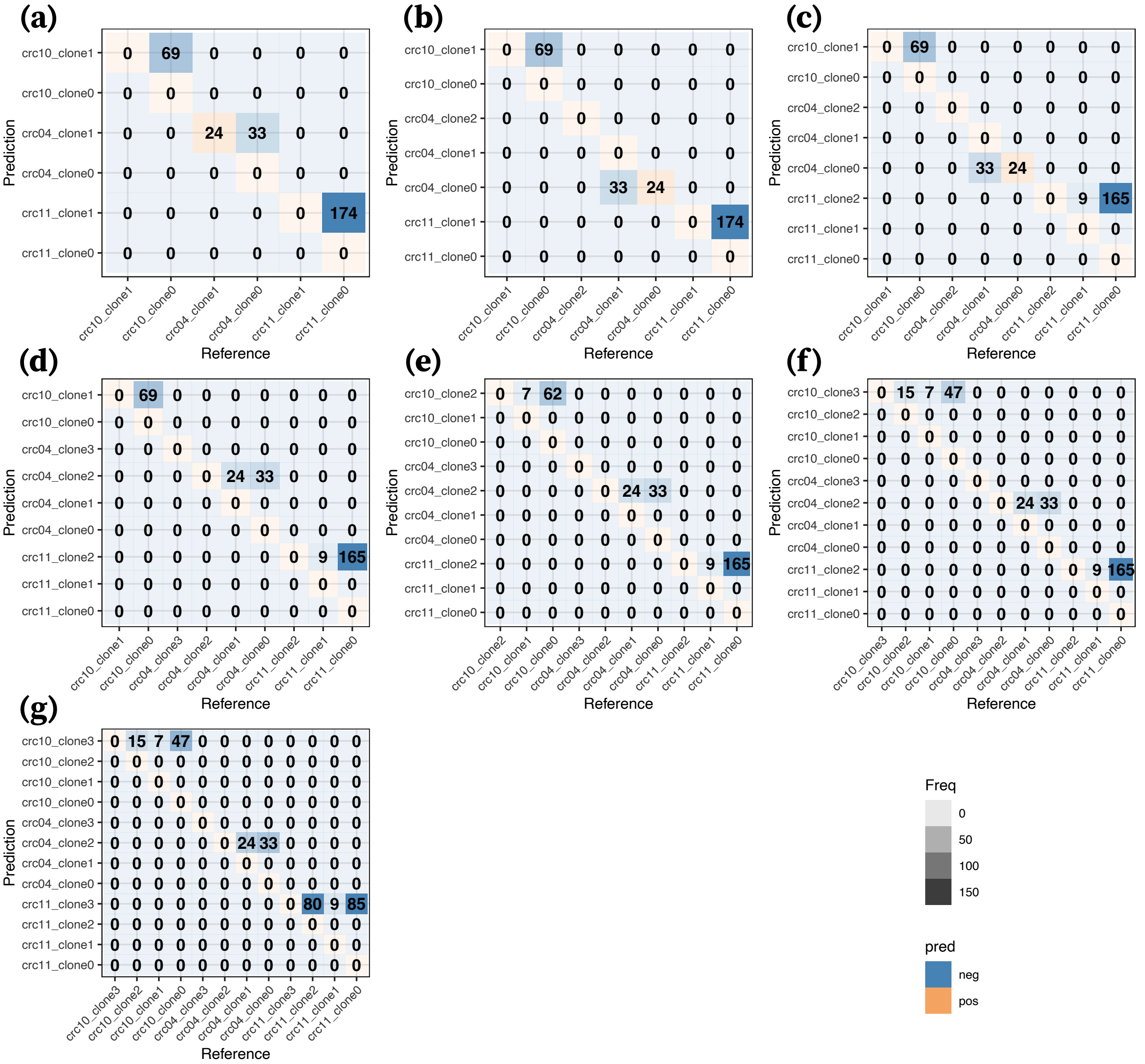

Figure S9: **clonealign results using agglomerative clustering method and the log-transformed data for clustering.**. Each panel shows the accuracy of clonealign on the three CRC patients in the form of a confusion matrix plot under the clustering resolution of six **(a)**, seven **(b)**, eight **(c)**, nine **(d)**, 10 **(e)**, 11 **(f)**, and 12 **(g)** clusters. The input to clonealign is the original data without the genes on X-chromosome. The data for clustering is the original log-transformed data. For each panel, we performed clonealign on each patient separately. Source data are provided as a Source Data file.

[Method] Seurat

[Clone method] Agglomerative

[Clone transformation] log

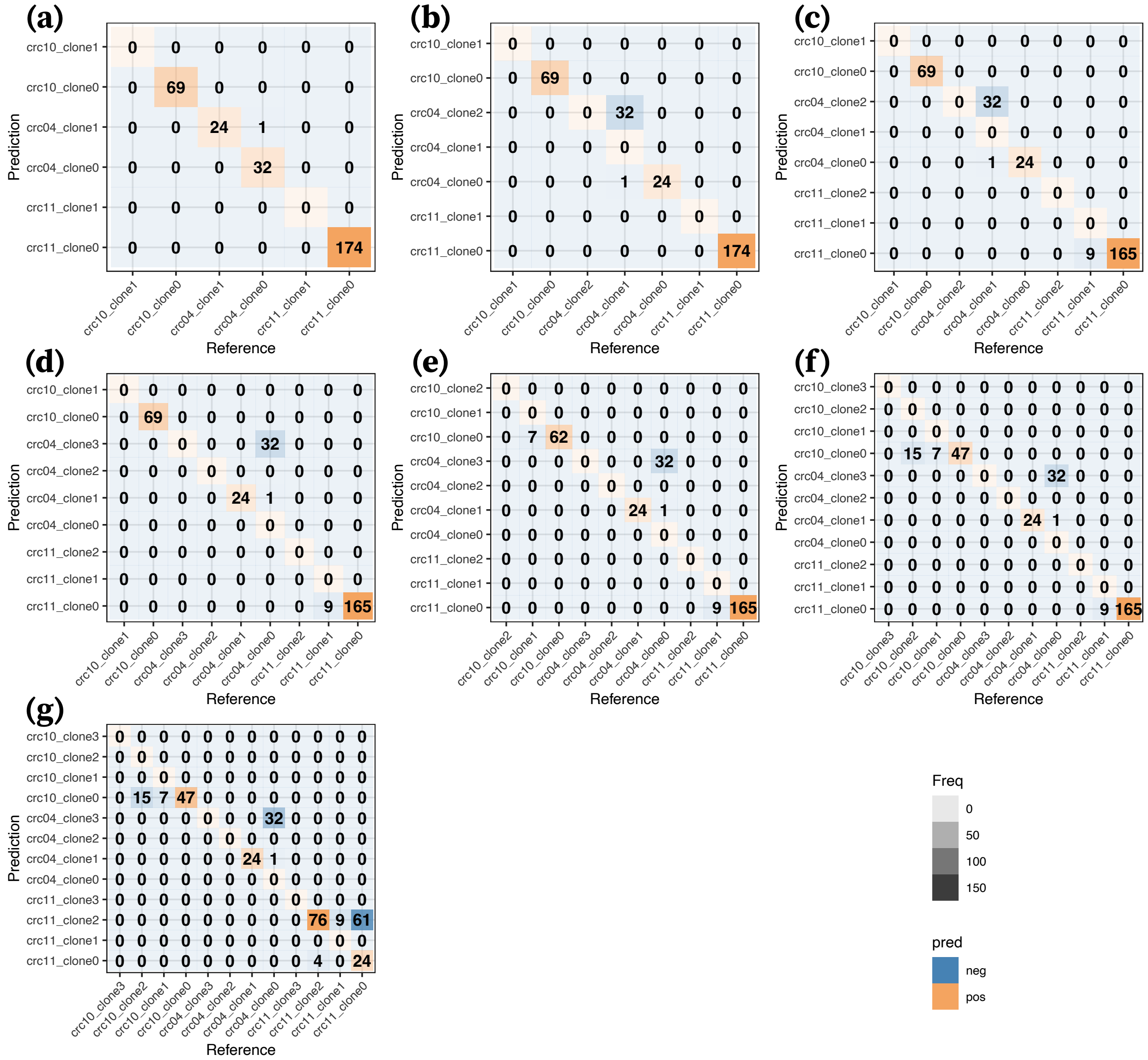

Figure S10: **Seurat results using agglomerative clustering method and the log-transformed data for clustering.** Each panel shows the accuracy of clonealign on the three CRC patients in the form of a confusion matrix plot under the clustering resolution of six **(a)**, seven **(b)**, eight **(c)**, nine **(d)**, 10 **(e)**, 11 **(f)**, and 12 **(g)** clusters. The input to Seurat is the log-transformed data with the top 2000 genes having been selected. The data for clustering is the log-transformed data. For each panel, we performed Seurat on each patient separately. Source data are provided as a Source Data file.

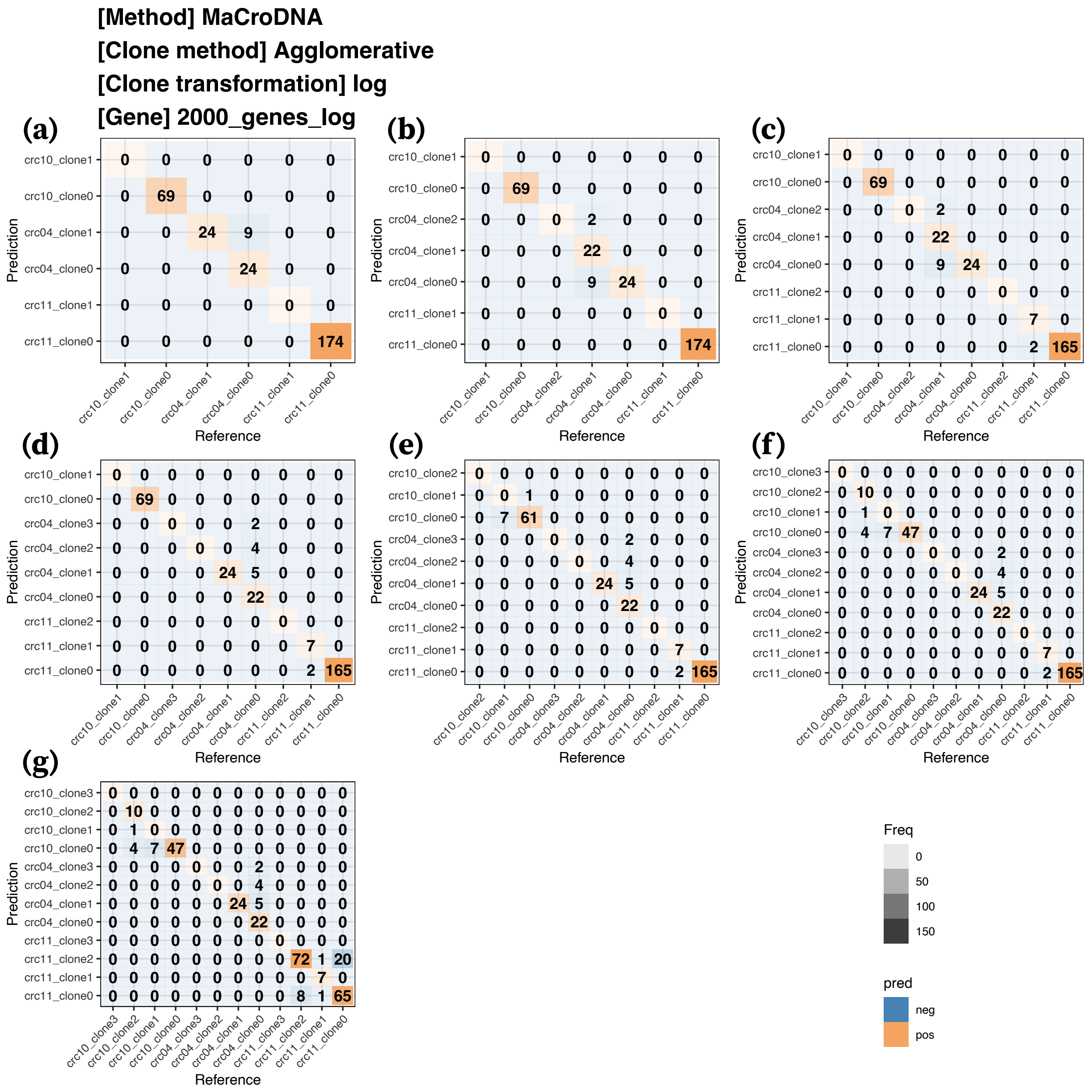

Figure S11: **MaCroDNA results using agglomerative clustering method and the log-transformed data for clustering.** Each panel shows the accuracy of clonealign on the three CRC patients in the form of a confusion matrix plot under the clustering resolution of six **(a)**, seven **(b)**, eight **(c)**, nine **(d)**, 10 **(e)**, 11 **(f)**, and 12 **(g)** clusters. The input to MaCroDNA is the same as the input for Seurat. The input is the log-transformed data of the original data. The top 2000 genes have been selected. The data for clustering is the log-transformed data. For each panel, we performed MaCroDNA on each patient separately. Source data are provided as a Source Data file.

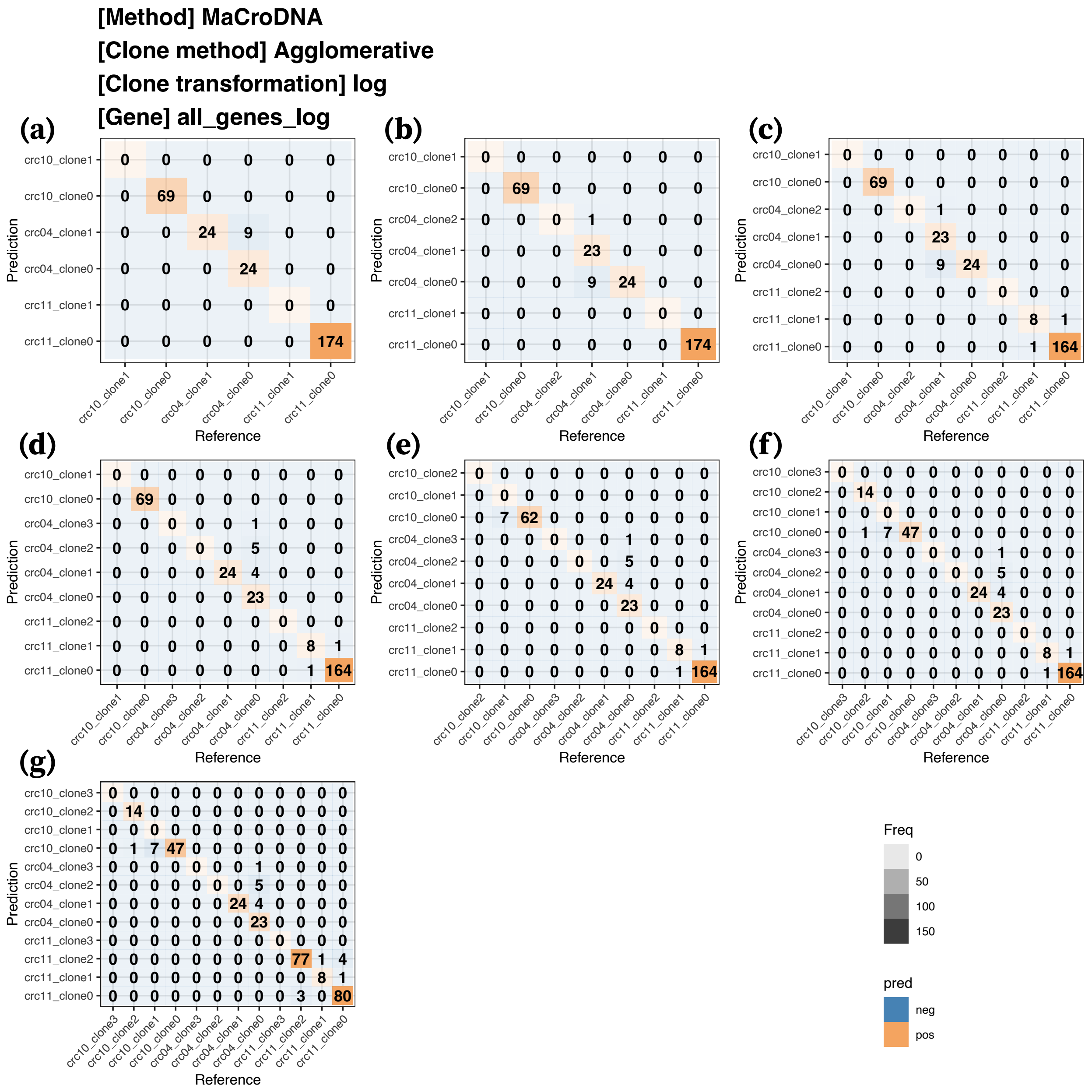

Figure S12: **MaCroDNA results using agglomerative clustering method and the log-transformed data for clustering.** Each panel shows the accuracy of clonealign on the three CRC patients in the form of a confusion matrix plot under the clustering resolution of six **(a)**, seven **(b)**, eight **(c)**, nine **(d)**, 10 **(e)**, 11 **(f)**, and 12 **(g)** clusters. The input to MaCroDNA is the log-transformed data, which is the same transformation applied to the input for Seurat, with the difference being that here, all genes are used. The data for clustering is the log-transformed data. For each panel, we performed MaCroDNA on each patient separately. Source data are provided as a Source Data file.

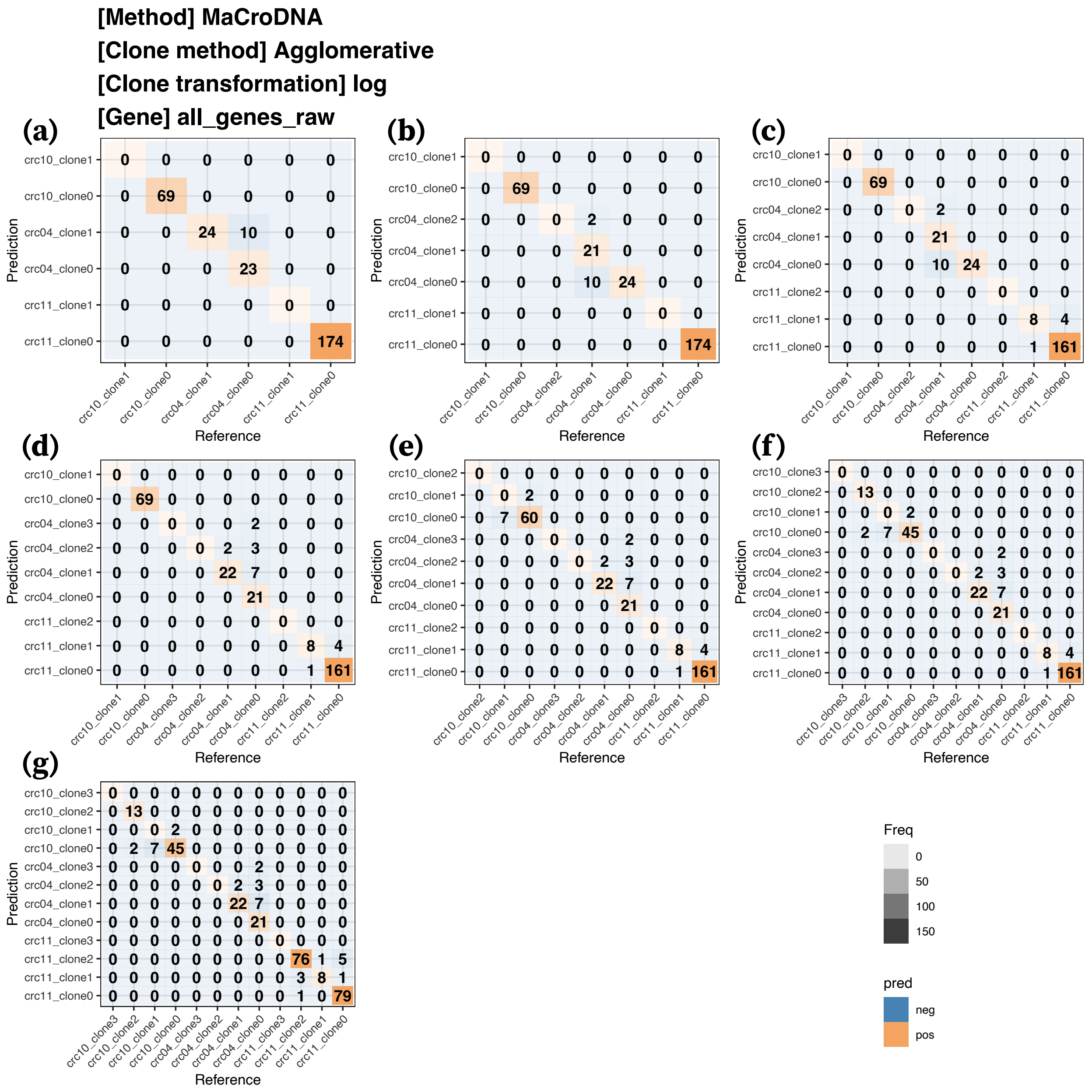

Figure S13: **MaCroDNA results using agglomerative clustering method and the log-transformed data for clustering.** Each panel shows the accuracy of clonealign on the three CRC patients in the form of a confusion matrix plot under the clustering resolution of six **(a)**, seven **(b)**, eight **(c)**, nine **(d)**, 10 **(e)**, 11 **(f)**, and 12 **(g)** clusters. The input to MaCroDNA is the original data with all the genes. The data for clustering is the log-transformed data. For each panel, we performed MaCroDNA on each patient separately. Source data are provided as a Source Data file.

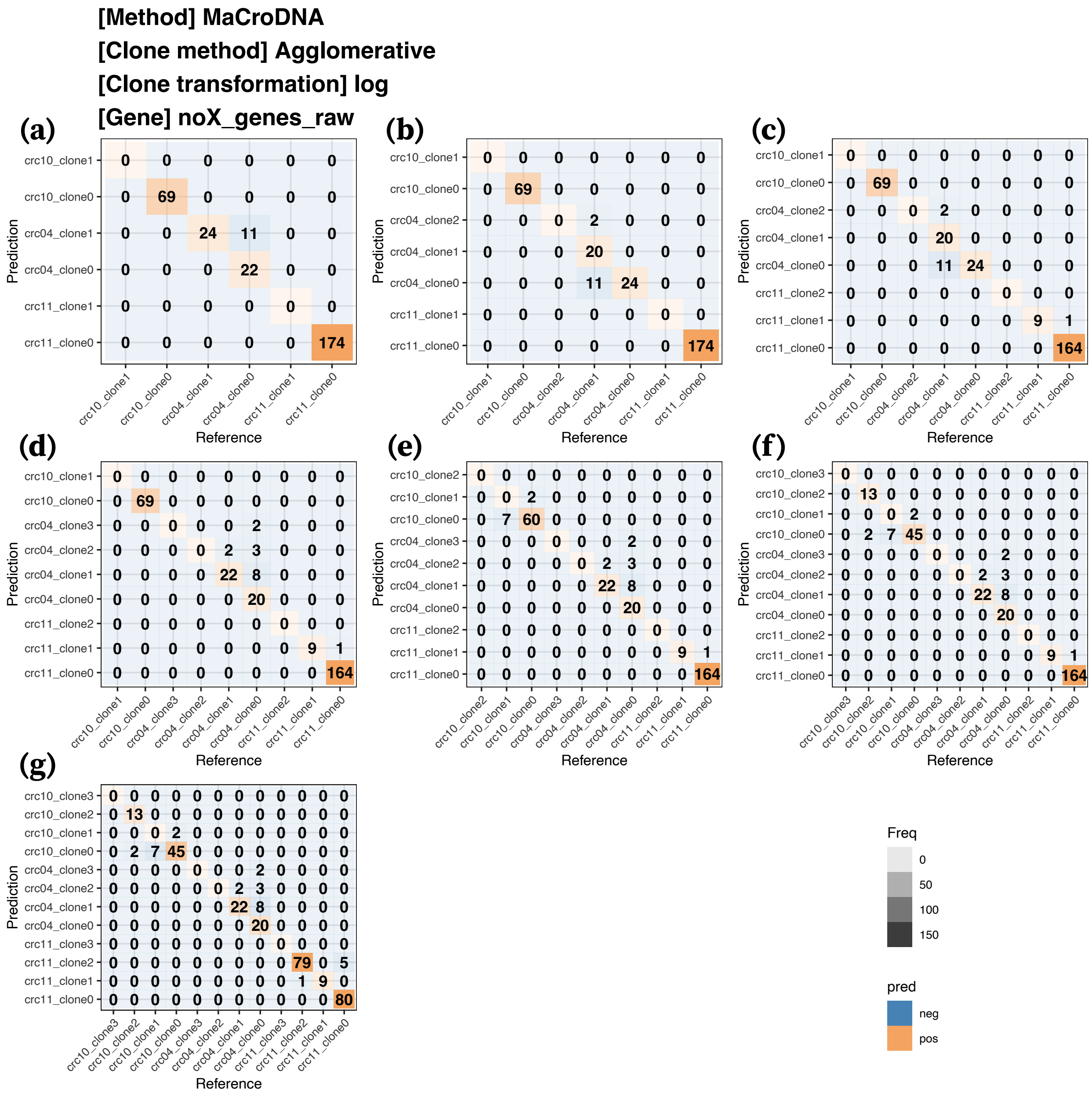

Figure S14: **MaCroDNA results using agglomerative clustering method and the log-transformed data for clustering.** Each panel shows the accuracy of clonealign on the three CRC patients in the form of a confusion matrix plot under the clustering resolution of six **(a)**, seven **(b)**, eight **(c)**, nine **(d)**, 10 **(e)**, 11 **(f)**, and 12 **(g)** clusters. The input to MaCroDNA is the original data without the genes on X-chromosome, the same as the input for clonealign. The data for clustering is the log-transformed data. For each panel, we performed MaCroDNA on each patient separately. Source data are provided as a Source Data file.

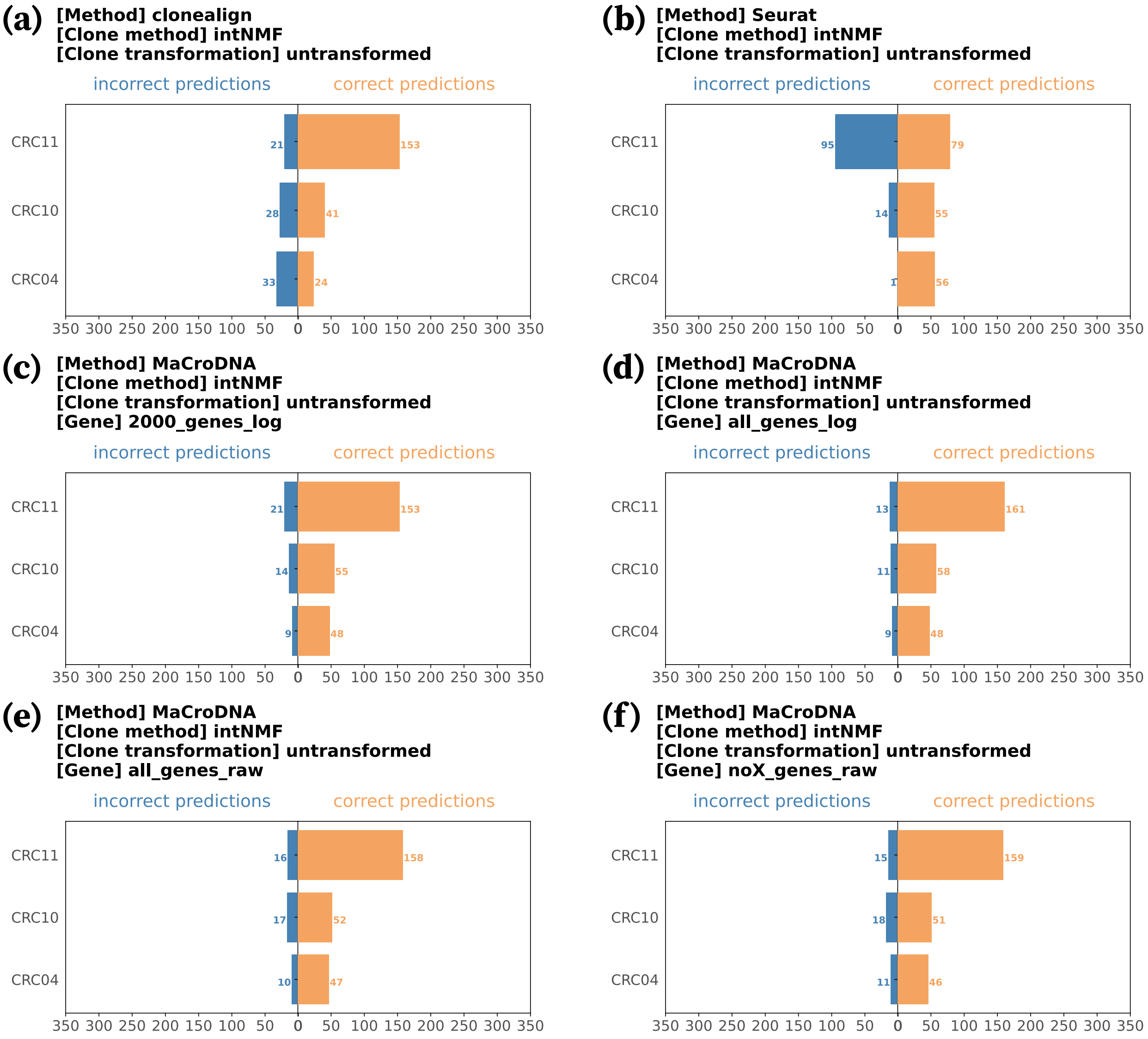

Figure S15: **Results of intNMF clustering method, using untransformed data for clustering.** Each panel is a mirror plot showing the accuracy of a method on the three CRC patients: **(a)** for clonealign, **(b)** for Seurat, and **(c-f)** for MaCroDNA with different preprocessing procedures on its input data. In panel **(a)**, the input of clonealign is the original data without the genes on X-chromosome. In panel **(b)**, the input of Seurat is the log-transformed data with the top 2000 genes having been selected. The input of MaCroDNA has four different preprocessing settings: in **(c)**, `2000_genes_log` is the same as the input of Seurat, in **(d)**, `all_genes_log` uses the same log-transformation for Seurat but all genes are used, in **(e)**, `all_genes_raw` is the original data with all genes, and in **(f)**, `noX_genes_raw` is the same as the input of clonealign: original values without the genes on X-chromosome. For each panel, we performed the corresponding method on each patient separately. Source data are provided as a Source Data file.

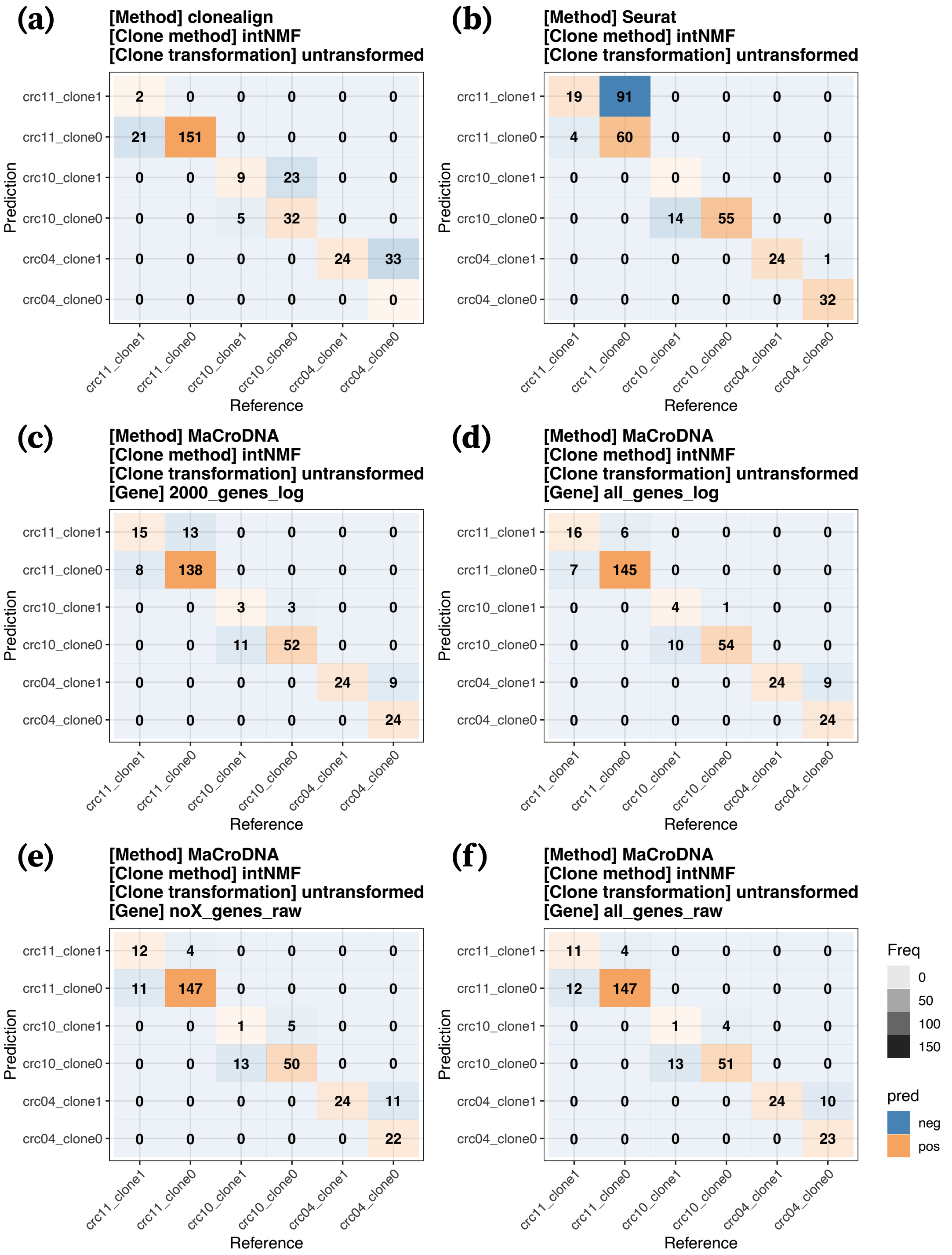

Figure S16: **Results of intNMF clustering method, using untransformed data for clustering.** Each panel shows the accuracy of clonealign on the three CRC patients in the form of a confusion matrix plot: **(a)** for clonealign, **(b)** for Seurat, and **(c-f)** for MaCroDNA with different preprocessing procedures on its input data. In panel **(a)**, the input of clonealign is the original data without the genes on X-chromosome. In panel **(b)**, the input of Seurat is the log-transformed data with the top 2000 genes having been selected. The input of MaCroDNA has four different settings: in **(c)**, `2000_genes_log` is the same as the input of Seurat, in **(d)**, `all_genes_log` uses the same log-transformation for Seurat but all genes are used, in **(e)**, `all_genes_raw` is the original data with all genes, and in **(f)**, `noX_genes_raw` is the same as the input of clonealign: original values without the genes on X-chromosome. For each panel, we performed the corresponding method on each patient separately. Source data are provided as a Source Data file.

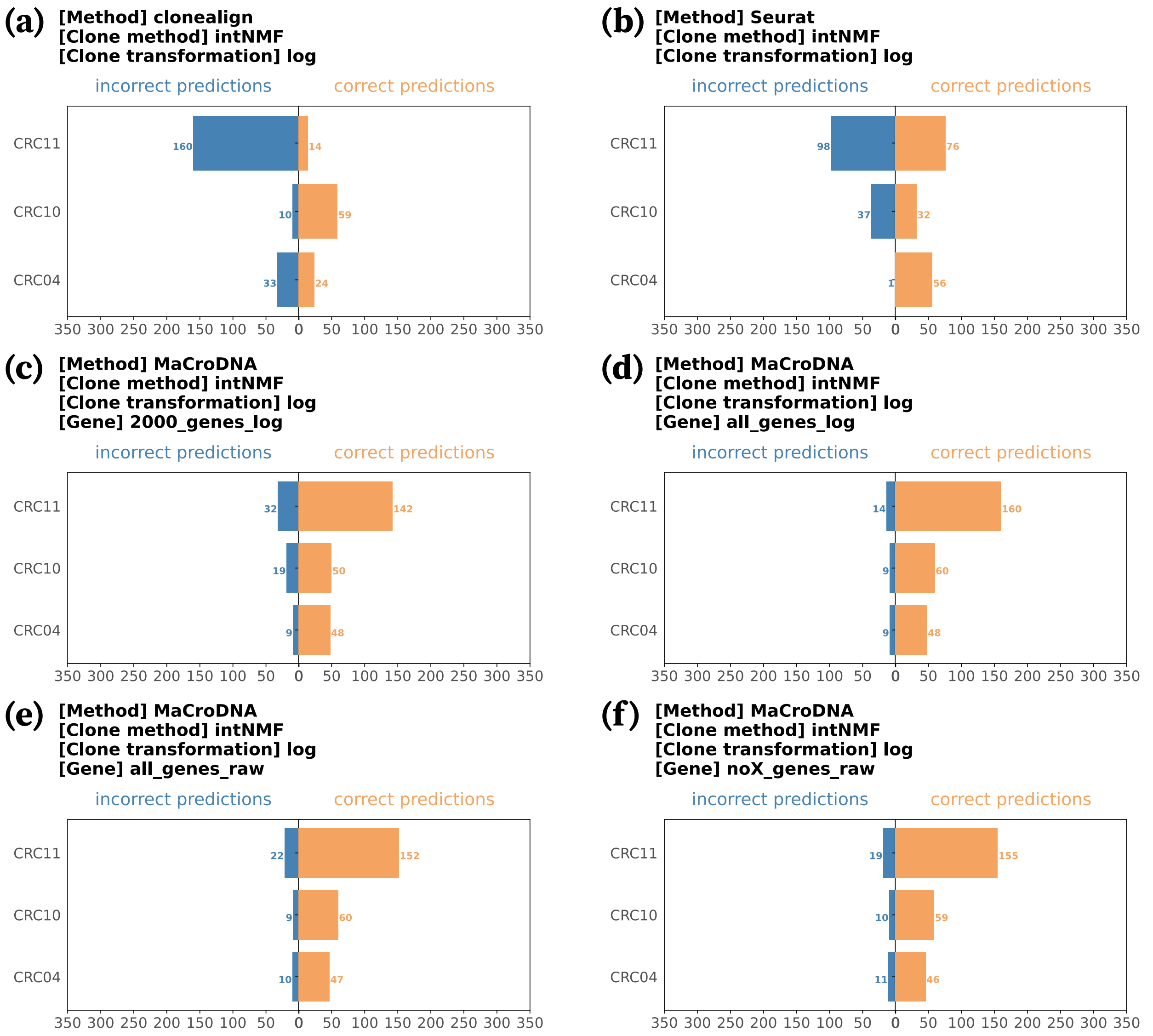

Figure S17: **Results of intNMF clustering method, using log-transformed data for clustering.** Each panel is a mirror plot showing the accuracy of a method on the three CRC patients: **(a)** for clonealign, **(b)** for Seurat, and **(c-f)** for MaCroDNA with different preprocessing procedures on its input data. In panel **(a)**, the input of clonealign is the original data without the genes on X-chromosome. In panel **(b)**, the input of Seurat is the log-transformed data with the top 2000 genes having been selected. The input of MaCroDNA has four different preprocessing settings: in **(c)**, `2000_genes_log` is the same as the input of Seurat, in **(d)**, `all_genes_log` uses the same log-transformation for Seurat but all genes are used, in **(e)**, `all_genes_raw` is the original data with all genes, and in **(f)**, `noX_genes_raw` is the same as the input of clonealign: original values without the genes on X-chromosome. For each panel, we performed the corresponding method on each patient separately. Source data are provided as a Source Data file.

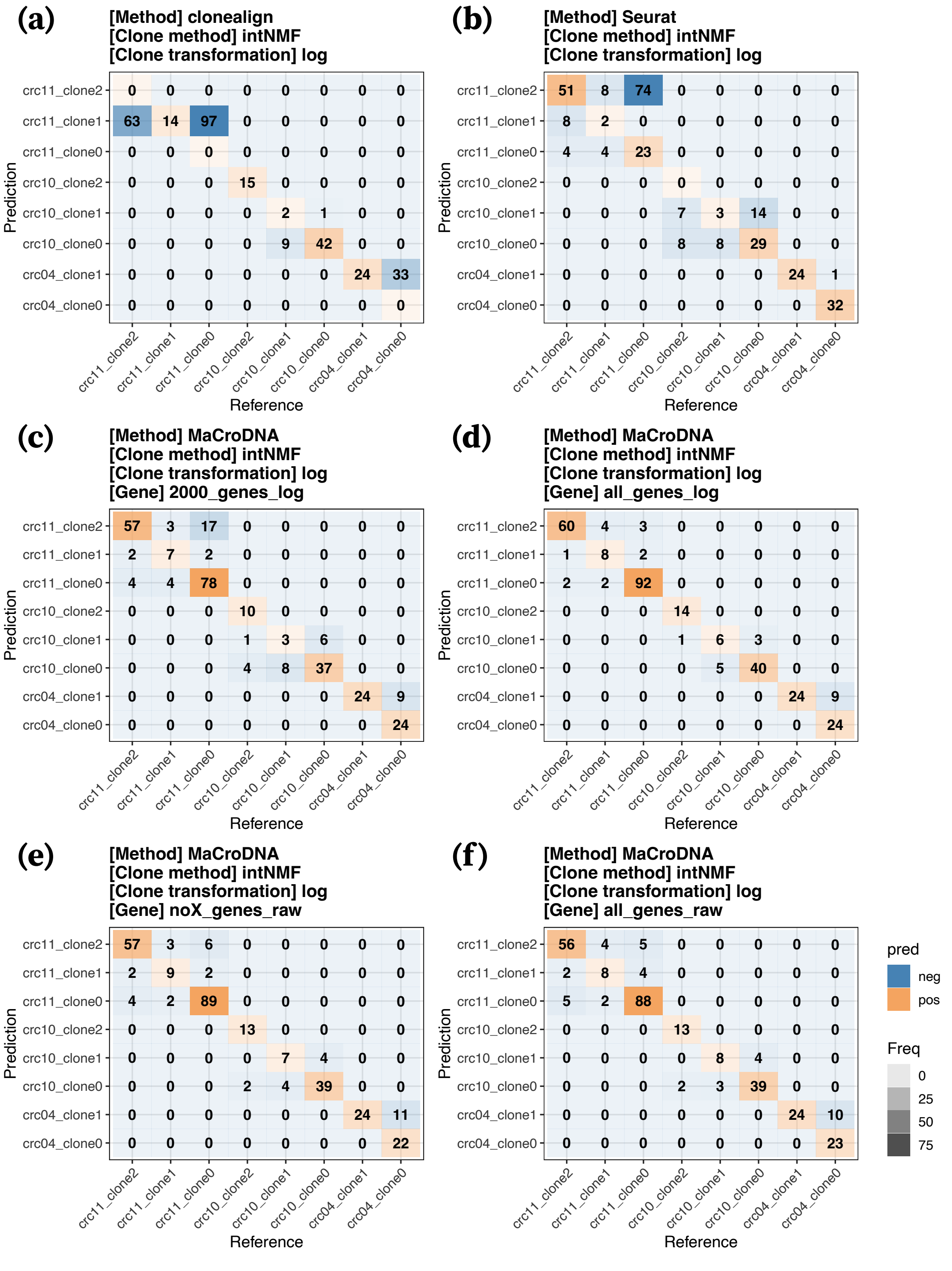

Figure S18: **Results of intNMF clustering method, using log-transformed data for clustering.** Each panel shows the accuracy of clonealign on the three CRC patients in the form of a confusion matrix plot: **(a)** for clonealign, **(b)** for Seurat, and **(c-f)** for MaCroDNA with different preprocessing procedures on its input data. In panel **(a)**, the input of clonealign is the original data without the genes on X-chromosome. In panel **(b)**, the input of Seurat is the log-transformed data with the top 2000 genes having been selected. The input of MaCroDNA has four different settings: in **(c)**, `2000_genes_log` is the same as the input of Seurat, in **(d)**, `all_genes_log` uses the same log-transformation for Seurat but all genes are used, in **(e)**, `all_genes_raw` is the original data with all genes, and in **(f)**, `noX_genes_raw` is the same as the input of clonealign: original values without the genes on X-chromosome. For each panel, we performed the corresponding method on each patient separately. Source data are provided as a Source Data file.

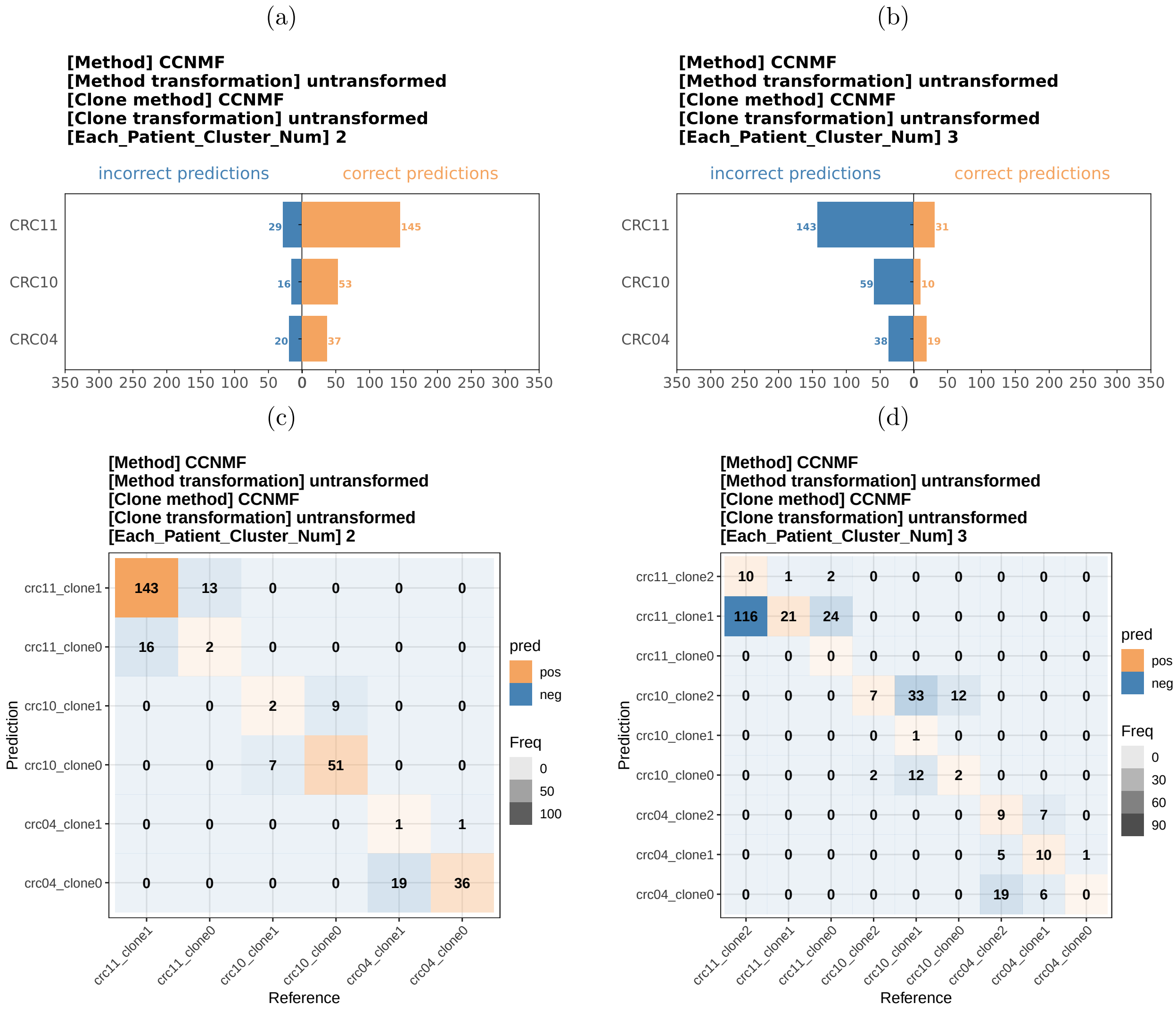

Prediction \ Reference (c)	crc11_clone1	crc11_clone0	crc10_clone1	crc10_clone0	crc04_clone1	crc04_clone0
crc11_clone1	143	13	0	0	0	0
crc11_clone0	16	2	0	0	0	0
crc10_clone1	0	0	2	9	0	0
crc10_clone0	0	0	7	51	0	0
crc04_clone1	0	0	0	0	1	1
crc04_clone0	0	0	0	0	19	36

Prediction \ Reference (d)	crc11_clone2	crc11_clone1	crc11_clone0	crc10_clone2	crc10_clone1	crc10_clone0	crc04_clone2	crc04_clone1	crc04_clone0
crc11_clone2	10	1	2	0	0	0	0	0	0
crc11_clone1	116	21	24	0	0	0	0	0	0
crc11_clone0	0	0	0	0	0	0	0	0	0
crc10_clone2	0	0	0	7	33	12	0	0	0
crc10_clone1	0	0	0	0	1	0	0	0	0
crc10_clone0	0	0	0	2	12	2	0	0	0
crc04_clone2	0	0	0	0	0	0	9	7	0
crc04_clone1	0	0	0	0	0	0	5	10	1
crc04_clone0	0	0	0	0	0	0	19	6	0

Figure S19: **Results of CCNMF using the original data.** Given a particular number of clusters by the user, CCNMF infers that number of clusters. Here, we performed two experiments, one with two clusters being inferred for each patient, and the other with three clusters for each patient (except for the number of clusters, the rest of CCNMF's input parameters were set as default). The input data are original values. The results of CCNMF with two clusters in each patient are shown in the forms of **(a)** mirror and **(c)** confusion matrix plots. CCNMF's results with three clusters in each patient are shown in the forms of **(b)** mirror and **(d)** confusion matrix plots. For each panel, we performed CCNMF on each patient separately. Source data are provided as a Source Data file.

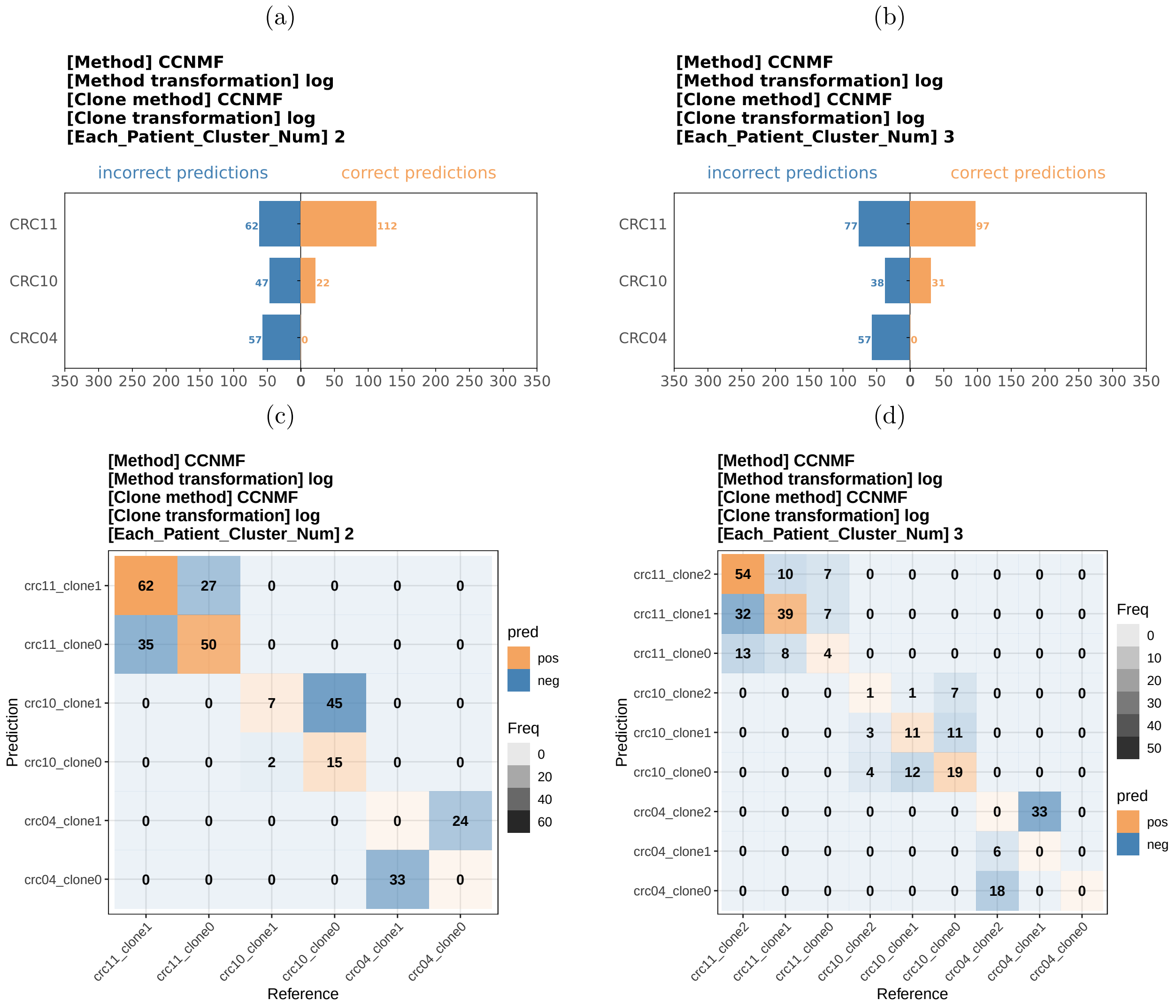

Figure S20: **Results of CCNMF using log-transformed data.** We performed two experiments, one with two clusters being inferred for each patient, and the other with three clusters for each patient. The input data are log-transformed. The results of CCNMF with two clusters in each patient are shown in the forms of **(a)** mirror and **(c)** confusion matrix plots. CCNMF's results with three clusters in each patient are shown in the forms of **(b)** mirror and **(d)** confusion matrix plots. For each panel, we performed CCNMF on each patient separately. Source data are provided as a Source Data file.

Table S1: K^* index for phylogenetic signal [1] was used to measure the contribution of genomic mutations to transcriptomic changes. For each gene, the p-value was estimated by a one-sided random permutation test with 999 repetitions to test K^* index against the null hypothesis of the gene expression values being randomly distributed in the phylogeny (sample size was number of DNA cells per biopsy: $n = 274$ for 16 (EAC), $n = 339$ for 20 (HGD1), $n = 117$ for 14 (HGD), $n = 312$ for 6 (HGD), $n = 362$ for 9 (NDBE), and $n = 342$ for 20 (CARD)). The following table contains the list of the COSMIC genes with statistically significant phylogenetic signal (i.e., p-value < 0.05 and $K^* > 1$) identified in the BE data set.

Patient ID (histology)	COSMIC genes with p-value < 0.05 and $K^* > 1$ [1]
16 (EAC)	*GNAS*[2], *BTG1, ATP1A1, B2M, DDX5, H3F3B, MSI2, PABPC1, EIF3E, HSP90AB1, ERBB3, ERBB2, HNRNPA2B1, H3F3A, RPL10, BTG2, MUC1, RPL5, SMARCE1, AKAP9*
20 (HGD1)	*PTPRC, LCP1, IKZF1, CCND2, MSN, PRKCB, TNFAIP3, FLNA, LCK, RHOH, PRF1, BTK, JAK3, PIM1, B2M, FNBP1, NFATC2, ITK, SEPT6, MUC1, ZNF331, PRDM1, SH2B3, IKZF3, GATA2, BLM, ERBB3, BCL2, MYH9, IDH2, LYL1, BCL11B, TAL1, KIAA1549, KIT, MALT1, CYLD, IL7R, CARD11*
14 (HGD)	*CXCR4, MUC16, SLC34A2, AXIN2, ZNRF3, SIRPA, ETV5, HMGA2, TP53, GATA1, SMAD2, CDKN1A, PRF1, CLP1, TFE3, TAL1, BCL2, CBFA2T3, BTK, WAS, ZNF331, PTPRT, FCGR2B, HLF, PTPN13, LYL1, KDSR, FAS, RARA, ITK, VAV1, JAK3, WWTR1, PIM1, SRGAP3, BRAF, PRKCB, POLD1, RMI2, FLI1, IRF4, IKZF1, RBM15, SNX29, CREB3L2, ID3, SEPT6, PRDM1, GATA3, SMAD4, BCL11B, COL1A1, JAZF1, LZTR1, CSF1R, MITF, ZCCHC8, LATS2, PLCG1, DDIT3, RHOH, CD28, CARD11, FNBP1, CDKN2C, NR4A3, ZMYM3, IL7R, HEY1, MNX1, FLNA, BAX, ELF4, MALT1, SMARCB1, SH2B3, PMS2, TBX3, NFATC2, MSN, MAFB, BLM, APOBEC3B, CHST11, STAT5B, ABL2, SMARCD1, CRTC3, SMAD3, XPC, FOXO1, KIT, FOXO4, EXT2, ERCC4, CEP89, CYLD, NCOA2, HOXA11, NOTCH1, TNFRSF14, NTHL1, DGCR8, RABEP1, CCND3, KMT2D, FANCE, SS18, PTPN6, NBEA, ACVR1B, NF2, PTPRC, FIP1L1, TLX1, ATF1, CPEB3, MYCL, CCND2*
6 (HGD)	*ERBB2, SMARCE1, MUC4, MSI2, CD74, KLF6, SUB1, FKBP9, NFIB, PABPC1, MACC1, ETNK1, NACA, TMPRSS2, HIF1A, KIF5B, HMGA1, SF3B1, ALDH2, MUC1, IDH1, ARHGEF10, KIT, HSP90AA1, LCP1, AKAP9, CTNNB1, NPM1, MECOM, MET, EIF4A2, BRD3, CTNNA1, FNBP1, ACSL3*
9 (NDBE)	*ERBB2, LASP1, ARID1B, SF3B1*
20 (CARD)	*MUC1, ALDH2, NDRG1, ELF3, SDC4, KLF4, CCND1*

[1] The names of the genes are sorted based on their corresponding K^* values in descending order.

[2] The gene names are italicized according to the organism-specific formatting guidelines for humans.

2 Supplementary Notes 1

Based on our experiment results, clonealign's performance was unsatisfactory when using agg-log clones, which are clones generated using the agglomerative clustering method on log-transformed data. When using agg-log clones, clonealign assigned all cells to a single clone. For example, all cells from patient CRC04 were assigned to crc04_clone2. Upon further examination of the data, as can be seen from Table S2, we discovered that in each patient, there is a copy-number-2 clone, where the copy number for every gene is 2. This is the clone that clonealign prefers assigning all the cells to. It is important to note that the input of clonealign is different from that of Seurat and MaCroDNA. The input of Seurat and MaCroDNA consists of three parts: a gene expression read count matrix, a cell copy number matrix, and the clone labels for all cells in the copy number matrix. However, clonealign only needs a gene expression read count matrix and a clone copy number matrix. The gene expression read count matrix is a cell-by-gene matrix containing the gene expression read count for each gene in each RNA cell. The cell copy number matrix is also a cell-by-gene matrix, and each entry is the copy number of one gene in one DNA cell. The clone copy number matrix, on the other hand, is a clone-by-gene matrix, with each entry representing the copy number of one gene in one clone. To obtain the copy number of one gene in one clone, we calculated the median value of that gene's copy numbers in all the cells in that clone. Even though the copy number is not 2 for each gene for each cell in that clone, the calculation of the median number results in the copy-number-2 clone, which serves as the input of clonealign.

After observing the results presented in Table S2, we conducted further analysis by excluding the copy-number-2 clones from the input. The results of this analysis are summarised in Table S3. As shown in the table, for CRC04, all the cells were still assigned to a single cluster with 100% probability, and the highest proportion of this clone is around 0.8. However, for CRC10 and CRC11, the assignment results were more balanced and the highest proportions are less than 0.65.

Taken together, Table S2 and Table S3 suggest that clonealign tends to assign cells to a single clone if it has a dominant copy number compared to the other clones. For simplicity, we denote a clone where every gene has the same copy number as a uniform-copy-number clone. If this clone has a significantly high proportion of its most common copy number, it is called a dominant-copy-

	Patient CRC04			
	crc04_clone0	crc04_clone1	**crc04_clone2**[a]	crc04_clone3
Most common copy number[b] (proportion)[c]	2 (0.549)	2 (0.792)	**2 (1.000)**	2 (0.502)
clonealign assignment [d] (probability)[e]	0 (0%)	0 (0%)	**93 (100%)**	0 (0%)

	Patient CRC10			
	crc10_clone0	crc10_clone1	crc10_clone2	**crc10_clone3**
Most common copy number (proportion)	2 (0.615)	2 (0.639)	2 (0.562)	**2 (1.000)**
clonealign assignment (probability)	0 (0%)	0 (0%)	0 (0%)	**85 (100%)**

	Patient CRC11			
	crc11_clone0	crc11_clone1	crc11_clone2	**crc11_clone3**
Most common copy number (proportion)	2 (0.554)	2 (0.551)	2 (0.547)	**2 (1.000)**
clonealign assignment (probability)	0 (0%)	0 (0%)	0 (0%)	**192 (100%)**

Table S2: The original clonealign results on clones generated from log-transformed data and agglomerative clustering method (agg-log clones).

[a]The columns shown in bold text indicate the clones in whose clonal copy number profiles, every gene's copy number is 2 (copy-number-2 clones).

[b]The most common copy number in the corresponding clone.

[c]The proportion of the most common copy number in the corresponding clone. "1" indicates that in the corresponding clone, each gene has the same copy number.

[d]The number of cells that clonealign assigned to the corresponding clone.

[e]The mean of the clonal assignment probability.

number clone. In Table S2, every patient has a copy-number-2 clone, which is also a uniform-copy-number clone. clonealign assigned all the cells to the copy-number-2 clones. In Table S3, CRC04 has a dominant-copy-number clone and clonealign still assigns all the cells to it. To further validate this observation, we conducted three sets of comprehensive experiments. Firstly, we evaluated clonealign's performance on other uniform-copy-number clones, where the copy number is not 2. Secondly, we assessed its effectiveness on dominant-copy-number clones by changing the proportion of the most common copy number. Lastly, we evaluated clonealign when there were two uniform-copy-number clones.

	Patient CRC04			
	crc04_clone0	**crc04_clone1**[a]	crc04_clone2	crc04_clone3
Most common copy number[b] (proportion)[d]	2 (0.549)	**2 (0.792)**	-[c] -	2 (0.502)
clonealign assignment [e] (probability)[f]	0 (0%)	**93 (100%)**	- -	0 (0%)

	Patient CRC10			
	crc10_clone0	crc10_clone1	crc10_clone2	crc10_clone3
Most common copy number (proportion)	2 (0.615)	2 (0.639)	2 (0.562)	- -
clonealign assignment (probability)	56 (65.9%)	14 (16.5%)	15 (17.6%)	- -

	Patient CRC11			
	crc11_clone0	crc11_clone1	crc11_clone2	crc11_clone3
Most common copy number (proportion)	2 (0.554)	2 (0.551)	2 (0.547)	- -
clonealign assignment (probability)	115 (59.9%)	36 (18.8%)	41 (21.4%)	- -

Table S3: clonealign performance on agg-log clones without copy-number-2 clones

[a]The column in bold text indicates the dominant-copy-number clone in patient CRC04.
[b]The most common copy number in the corresponding clone.
[c]Denotes the exclusion of this clone in the experiment.
[d]The proportion of the most common copy number in the corresponding clone. “1” indicates that in the corresponding clone, each gene has the same copy number.
[e]The number of cells that clonealign assigned to the corresponding clone.
[f]The mean of the clonal assignment probability.

2.1 Experiment 1: investigating clonealign's behavior with single uniform-copy-number clones

In our first experiment, we aimed to determine if clonealign's tendency to assign cells to a single clone is specific to copy-number-2 clones, or if it occurs for any uniform-copy-number clone. We tested both copy number duplication and deletion scenarios in this experiment. According to the clonealign paper, the default range of copy number is from 1 to 5, so we chose 1 and 5 as deletion and duplication values, respectively. For the copy number deletion scenario, we removed the existing copy-number-2 clone for each patient and added a new copy-number-1 clone where the copy number for each gene is 1. Other clones remained unchanged. For the copy number duplication case, we similarly removed the existing copy-number-2 clone and added a new copy-number-5 clone.

clonealign was tested on these two scenarios separately.

Our results, shown in Tables S4 and S5, indicate that clonealign tends to assign cells to any uniform-copy-number clone regardless of the magnitude of the copy number. For CRC04 and CRC11, clonealign assigned all the cells to the copy-number-1 clone or the copy-number-5 clone. For CRC10, clonealign assigned all the cells to the copy-number-5 clone and most cells to the copy-number-1 clone. Overall, these results indicate that clonealign has a strong preference for any uniform-copy-number clone.

	Patient CRC04				
	crc04_clone0	crc04_clone1	crc04_clone2	crc04_clone3	**copy_number_1**[a]**.**
Most common copy number[b] (proportion)[d]	2 (0.549)	2 (0.792)	-[c] -	2 (0.502)	**1 (1.000)**
clonealign assignment [e] (probability)[f]	0 (0%)	0 (0%)	- -	0 (0%)	**93 (100%)**

	Patient CRC10				
	crc10_clone0	crc10_clone1	crc10_clone2	crc10_clone3	**copy_number_1**
Most common copy number (proportion)	2 (0.615)	2 (0.639)	2 (0.562)	- -	**1 (1.000)**
clonealign assignment (probability)	1 (1.2%)	0 (0%)	1 (1.2%)	- -	**83 (97.6%)**

	Patient CRC11				
	crc11_clone0	crc11_clone1	crc11_clone2	crc11_clone3	**copy_number_1**
Most common copy number (proportion)	2 (0.554)	2 (0.551)	2 (0.547)	- -	**1 (1.000)**
clonealign assignment (probability)	0 (0%)	0 (0%)	0 (0%)	- -	**192 (100%)**

Table S4: Experiment 1 clonealign performance on copy-number-1 clones

[a]The columns in bold text indicate the uniform-copy-number clones, with a copy number of 1 for each gene
[b]The most common copy number in the corresponding clone.
[c]Denotes the exclusion of this clone in the experiment.
[d]The proportion of the most common copy number in the corresponding clone. "1" indicates that in the corresponding clone, each gene has the same copy number.)
[e]The number of cells that clonealign assigned to the corresponding clone.
[f]The mean of the clonal assignment probability.

2.2 Experiment 2: investigating clonealign's behavior with dominant-copy-number clones

In this experiment, we aimed to investigate clonealign's behavior when dealing with dominant-copy-number clones. A dominant-copy-number clone is defined as a clone that has a significantly

	Patient CRC04				
	crc04_clone0	crc04_clone1	crc04_clone2	crc04_clone3	**copy_number_5**[a]**.**
Most common copy number[b]<br>(proportion)[d]	2<br>(0.549)	2<br>(0.792)	-[c]<br>-	2<br>(0.502)	**1**<br>**(1.000)**
clonealign assignment [e]<br>(probability)[f]	0<br>(0%)	0<br>(0%)	-<br>-	0<br>(0%)	**93**<br>**(100%)**

	Patient CRC10				
	crc10_clone0	crc10_clone1	crc10_clone2	crc10_clone3	**copy_number_5**
Most common copy number<br>(proportion)	2<br>(0.615)	2<br>(0.639)	2<br>(0.562)	-<br>-	**1**<br>**(1.000)**
clonealign assignment<br>(probability)	0<br>(0%)	0<br>(0%)	0<br>(0%)	-<br>-	**85**<br>**(100%)**

	Patient CRC11				
	crc11_clone0	crc11_clone1	crc11_clone2	crc11_clone3	**copy_number_5**
Most common copy number<br>(proportion)	2<br>(0.554)	2<br>(0.551)	2<br>(0.547)	-<br>-	**1**<br>**(1.000)**
clonealign assignment<br>(probability)	0<br>(0%)	0<br>(0%)	0<br>(0%)	-<br>-	**192**<br>**(100%)**

Table S5: Experiment 1 clonealign performance on copy-number-5 clones

[a] The columns in bold text indicate the uniform-copy-number clones, with a copy number of 5 for each gene

[b] The most common copy number in the corresponding clone.

[c] Denotes the exclusion of this clone in the experiment.

[d] The proportion of the most common copy number in the corresponding clone. "1" indicates that in the corresponding clone, each gene has the same copy number.)

[e] The number of cells that clonealign assigned to the corresponding clone.

[f] The mean of the clonal assignment probability.

high proportion of its most common copy number. This differs from a uniform-copy-number clone, where all genes have the same copy number. To investigate clonealign's behavior with dominant-copy-number clones, we created three tests using dominant-copy-number clones with different proportions. We chose proportions of 0.9, 0.8 and 0.7 to represent high, moderate, and low levels of dominance, respectively. In both tests, the existing copy-number-2 clones were excluded, and a new clone with 2 as the dominant copy number was added. In the first test, we randomly selected 0.9 of the genes and gave them copy number 2. For the rest of the genes, the copy number was randomly selected from 1,3,4,5. For the second test, we reduced the proportion to 0.8 and for the third test to 0.7, following the same procedure as in the first test. We then ran clonealign on these tests.

Our results indicate that clonealign's preference for dominant-copy-number clones decreases as the dominance level decreases. Table S6 shows the results of our first test, where we used a

proportion of 0.9. In this test, clonealign assigned almost all the cells to the dominant-copy-number clones for patients CRC10 and CRC11. For patient CRC04, there are two dominant-copy-number clones, and clonealign assigned more cells to the clone with a higher proportion. Table S7 shows the results of our second test, where we reduced the proportion to 0.8. For patient CRC04, clonealign assigned all the cells to the two dominant-copy-number clones. For patient CRC10, the assignment is more balanced. For patient CRC11, the dominant clone has the most cells. Table S8 shows the results of our third test, where we further reduced the proportion to 0.7. For patient CRC04, most cells were assigned to the dominant-copy-number clone with moderate dominance. For patient CRC11, although the dominance level is low for the dominant-copy-number clone, about half of the cells were assigned to it. For patient CRC10, the dominant-copy-number clone loses its dominant place.

In summary, these findings suggest that clonealign's performance with dominant-copy-number clones is influenced by the level of dominance and that it has a strong preference for dominant-copy-number clones with high or moderate levels of dominance.

2.3 Experiment 3: investigating clonealign's behavior with multiple uniform-copy-number clones

In addition to the above experiments, we also investigated what will happen if there are two uniform-copy-number clones. This differs from our first experiment, where we investigated clonealign's behavior with a single uniform-copy-number clone. To conduct this experiment, we added a copy-number-5 clone on top of the original clone and ran clonealign three times with different random seeds to see if the results would change.

As shown in Table S9, clonealign only assigned cells to the two uniform-copy-number clones, with one of these clones taking up the majority of the cells. Furthermore, we observed that clonealign performed inconsistently across multiple tests, with its choice between the uniform-copy-number clones appearing to be random.

These findings suggest that when there are multiple uniform-copy-number clones, clonealign may choose one of these clones to assign the majority of cells and assign the rest of the cells to the

	Patient CRC04				
	crc04_clone0	**crc04_clone1** [a]	crc04_clone2	crc04_clone3	**proportion_0.9**
Most common copy number[b] (proportion)[d]	2 (0.549)	**2 (0.792)**	-[c] -	2 (0.502)	**2 (0.900)**
clonealign assignment [e] (probability)[f]	0 (0%)	**23 (24.7%)**	- -	0 (0%)	**70 (75.3%)**

	Patient CRC10				
	crc10_clone0	crc10_clone1	crc10_clone2	crc10_clone3	**proportion_0.9**
Most common copy number (proportion)	2 (0.615)	2 (0.639)	2 (0.562)	- -	**2 (0.900)**
clonealign assignment (probability)	2 (2.4%)	1 (1.2%)	2 (2.4%)	- -	**80 (94.1%)**

	Patient CRC11				
	crc11_clone0	crc11_clone1	crc11_clone2	crc11_clone3	**proportion_0.9**
Most common copy number (proportion)	2 (0.554)	2 (0.551)	2 (0.547)	- -	**2 (0.900)**
clonealign assignment (probability)	2 (1.0%)	0 (0%)	0 (0%)	- -	**190 (99.0%)**

Table S6: Experiment 2 clonealign performance on dominant-copy-number clones with proportion 0.9

[a]The columns in bold text indicate the dominant-copy-number clones.

[b]The most common copy number in the corresponding clone.

[c]Denotes the exclusion of this clone in the experiment.

[d]The proportion of the most common copy number in the corresponding clone. “1” indicates that in the corresponding clone, each gene has the same copy number.)

[e]The number of cells that clonealign assigned to the corresponding clone.

[f]The mean of the clonal assignment probability.

other uniform-copy-number clone. However, its choice between these clones may be inconsistent and unpredictable.

2.4 Investigation of the cause of clonealign's performance

After observing clonealign's strong preference for uniform-copy-number clones and dominant-copy-number clones, we decided to investigate the reason for its performance. clonealign applies mean-field variational Bayes to obtain the variational approximation. After running the code line by line, we found out that clonealign assigns all cells to the uniform-copy-number clones at the first iteration. However, due to a lack of detailed ELBO for the variational approximation, we were unable to determine why this happens.

Despite these challenges, our investigation provided valuable insights into clonealign's behavior

	Patient CRC04				
	crc04_clone0	**crc04_clone1**[a]	crc04_clone2	crc04_clone3	**proportion_0.8**
Most common copy number[b] (proportion)[d]	2 (0.549)	**2 (0.792)**	-[c] -	2 (0.502)	**2 (0.800)**
clonealign assignment [e] (probability)[f]	0 (0%)	**80 (86.0%)**	- -	0 (0%)	**13 (14.0%)**

	Patient CRC10				
	**crc10_clone0**	crc10_clone1	crc10_clone2	crc10_clone3	**proportion_0.8**
Most common copy number (proportion)	**2 (0.615)**	2 (0.639)	2 (0.562)	- -	**2 (0.800)**
clonealign assignment (probability)	**30 (35.3%)**	11 (12.9%)	12 (14.1%)	- -	**32 (37.6%)**

	Patient CRC11				
	crc11_clone0	crc11_clone1	crc11_clone2	crc11_clone3	**proportion_0.8**
Most common copy number (proportion)	2 (0.554)	2 (0.551)	2 (0.547)	- -	**2 (0.800)**
clonealign assignment (probability)	6 (3.1%)	7 (3.6%)	5 (2.6%)	- -	**174 (90.6%)**

Table S7: Experiment 2 clonealign performance on dominant-copy-number clones with proportion 0.8

[a]The columns in bold text indicate the clones to which clonealign assigned the majority of the cells.
[b]The most common copy number in the corresponding clone.
[c]Denotes the exclusion of this clone in the experiment.
[d]The proportion of the most common copy number in the corresponding clone. "1" indicates that in the corresponding clone, each gene has the same copy number.)
[e]The number of cells that clonealign assigned to the corresponding clone.
[f]The mean of the clonal assignment probability.

and highlighted the need for further research to fully understand its performance with uniform-copy-number clones and dominant-copy-number clones.

2.5 Investigating clonealign's accuracy by adding pseudo-count values to the inputs

To further investigate the effect of data preprocessing on the accuracy of clonealign, when preparing the input data (which we denoted as `noX_genes_raw`), instead of removing the genes with zero absolute copy number in at least one cell, we added pseudo-count value of 1 to all absolute copy number values. Next, we performed clonealign on the new data to compare the results with that of the main preprocessing scheme. Fig. S21 illustrates the accuracy of clonealign for both preprocessing procedures, the results from the removal of genes with at least one zero copy number value are

	Patient CRC04				
	crc04_clone0	**crc04_clone1**[a]	crc04_clone2	crc04_clone3	proportion_0.7
Most common copy number[b] (proportion)[d]	2 (0.549)	**2 (0.792)**	-[c] -	2 (0.502)	2 (0.700)
clonealign assignment [e] (probability)[f]	0 (0%)	**91 (97.8%)**	- -	0 (0%)	2 (2.2%)
	Patient CRC10				
	**crc10_clone0**	crc10_clone1	crc10_clone2	crc10_clone3	proportion_0.7
Most common copy number (proportion)	**2 (0.615)**	2 (0.639)	2 (0.562)	- -	2 (0.700)
clonealign assignment (probability)	**43 (50.6%)**	19 (22.4%)	16 (18.8%)	- -	7 (8.2%)
	Patient CRC11				
	crc11_clone0	crc11_clone1	crc11_clone2	crc11_clone3	**proportion_0.7**
Most common copy number (proportion)	2 (0.554)	2 (0.551)	2 (0.547)	- -	**2 (0.700)**
clonealign assignment (probability)	36 (18.7%)	23 (12.0%)	22 (11.5%)	- -	**111 (57.8%)**

Table S8: Experiment 2 clonealign performance on dominant-copy-number clones with proportion 0.7

[a]The columns in bold text indicate the clone to which clonealign assigned the majority of the cells.
[b]The most common copy number in the corresponding clone.
[c]Denotes the exclusion of this clone in the experiment.
[d]The proportion of the most common copy number in the corresponding clone. "1" indicates that in the corresponding clone, each gene has the same copy number.)
[e]The number of cells that clonealign assigned to the corresponding clone.
[f]The mean of the clonal assignment probability.

denoted by `clonealign_rm0`, and those from the addition of the pseudo-count value of 1 to all entries are denoted by `clonealign_add1`. As shown in Fig. S21, adding the pseudo-count values did not improve the accuracy of clonealign and therefore, we decided to present the results of clonealign from the removal of the genes with at least one zero copy number value as the main results of clonealign.

	Patient CRC04				
	crc04_clone0	crc04_clone1	**crc04_clone2**[a]	crc04_clone3	**copy_number_5**
Most common copy number[b] (proportion)[c]	2 (0.549)	2 (0.792)	**2 (1.000)**	2 (0.502)	**5 (1.000)**
clonealign assignment [d] (probability)[f]	0 (0%)	0 (0%)	**0/0/93**[e] **(31.6%/21.7%/93.6%)**	0 (0%)	**93/93/0 (68.4%/78.3%/6.4%)**

	Patient CRC10				
	crc10_clone0	crc10_clone1	crc10_clone2	**crc10_clone3**	**copy_number_5**
Most common copy number (proportion)	2 (0.615)	2 (0.639)	2 (0.562)	**2 (1.000)**	**5 (1.000)**
clonealign assignment (probability)	0 (0%)	0 (0%)	0 (0%)	**0/85/0 (19.8%/85.0%/29.0%)**	**85/0/85 (80.2%/15.0%/71.0%)**

	Patient CRC11				
	crc11_clone0	crc11_clone1	crc11_clone2	**crc11_clone3**	**copy_number_5**
Most common copy number (proportion)	2 (0.615)	2 (0.639)	2 (0.562)	**2 (1.000)**	**5 (1.000)**
clonealign assignment (probability)	0 (0%)	0 (0%)	0 (0%)	**189/0/191 (62.1%/15.9%/64.8%)**	**3/192/1 (37.9%/84.1%/35.2%)**

Table S9: Experiment 3 clonealign performance on two uniform-copy-number clones.

[a]The columns in bold text indicate the uniform-copy-number clones.

[b]The most common copy number in the corresponding clone.

[c]The proportion of the most common copy number in the corresponding clone. “1” indicates that in the corresponding clone, each gene has the same copy number.)

[d]The number of cells that clonealign assigned to the corresponding clone.

[e]x/x/x denotes the results from three repeated experiments with different random seed

[f]The mean of the clonal assignment probability

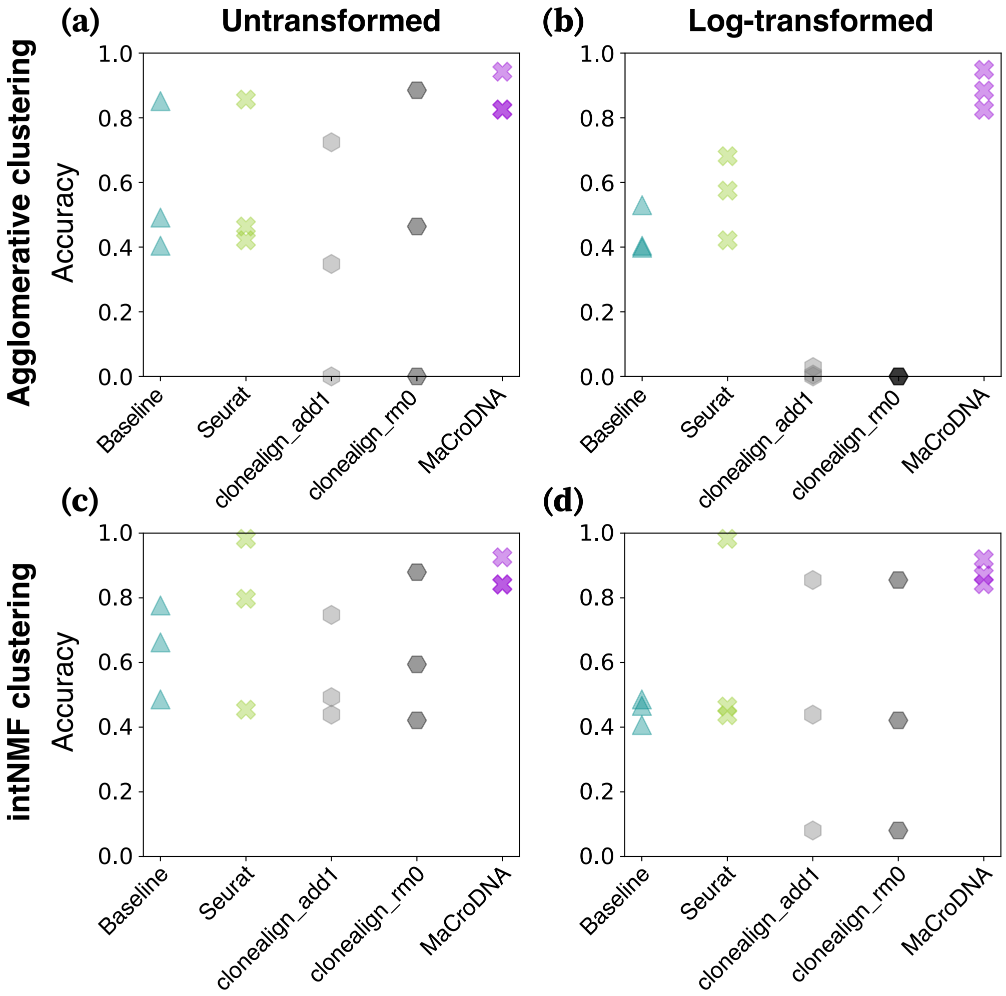

Figure S21: **clonealign's clonal assignment accuracy with two preprocessing procedures, along with the accuracy of the other methods.** Each panel displays the methods' accuracy across the three patients when the clustering algorithm and the input data to the clustering algorithm were **(a)** the agglomerative clustering, and the original data, **(b)** the agglomerative clustering and the log-transformed data, **(c)** the intNMF clustering and the original data, and **(d)** intNMF and the log-transformed data. clonealign's results when removing the genes with at least one zero copy number are tagged with `clonealign_rm0`, and those with adding pseudo-count values of 1 to all copy number values are tagged with `clonealign_add1`. Our results show that the removal of genes with at least one zero copy number yields better results compared to adding pseudo-count values. Source data are provided as a Source Data file.

3 Supplementary Notes 2

3.1 Investigating the effect of imbalance in clonal proportions across modalities in CRC patients

Since our method is based on the assumption that the proportion of clones across the two modalities is similar to each other, we investigated the effect of deviation from this assumption on the performance of MaCroDNA on the CRC data set. In particular, for each CRC patient, we performed two experiments that involved resampling the original data sets, resulting in variations in clonal proportions between scDNA-seq and scRNA-seq data. To achieve this goal, we used the two following techniques:

- **Random removal**: we randomly selected a limited number of scDNA-seq cells with joint DNA-RNA information and removed them from the DNA data as we inputted the RNA and DNA data to MaCroDNA.
- **Clonal proportion resampling**: we drew new proportions for the DNA clones and resampled the scDNA-seq cells with replacement to achieve the randomly drawn clonal proportions.

In the following, we elaborate on the details of these experiments.

3.1.1 Random removal of scDNA-seq cells from DNA data

In this experiment, for each CRC patient, we performed the following test:

1. From the scDNA-seq cells whose joint RNA information is available, randomly select 10 cells and remove them from the original DNA data.
2. Run MaCroDNA on the new data set and measure the accuracy of clonal assignments only for the scRNA-seq cells whose actual scDNA-seq pairs were removed from the DNA data in the previous step.

In this scenario, a scRNA-seq cell whose true pair is removed from the DNA data is correctly assigned to a clone if the clone ID is the same as the clone ID of its scDNA-seq pair. We repeated the

above test 10,000 times on the preprocessed CRC data named `all_genes_log` with different clustering settings. For each clustering setting, we used the highest clustering resolution available, that is, four clusters per patient when using agglomerative clustering and the optimal number of clusters when using the intNMF clustering algorithm. First, we looked into the accuracy of the scRNA-seq cells' clonal assignment in the presence and absence of their true scDNA-seq pairs. Fig. S22 displays the accuracy measurements under these two conditions for all patients and clustering techniques. The box plots show that the accuracy of clonal assignments decreases when the true scDNA-seq cells are absent in the DNA data.

Furthermore, we aimed to measure the extent to which the accuracy changed due to the removal of ten randomly sampled scDNA-seq cells in each repetition of the random removal test. To achieve this, for each test, we subtracted the accuracy of clonal assignments in the absence of true pairs from that in their presence. Fig. S23 shows this change in accuracy for all CRC patients under different clustering techniques. As shown in the figure, in most cases, the median of changes ranged between 0% to 10%, i.e., zero or one more wrong assignment occurred as the consequence of the removal of the true pairs. Interestingly, we observed negative values in the lower tail of the distributions meaning the clonal assignment accuracy increased in some rare cases.

3.1.2 Resampling clonal proportions in DNA data

The previous experiment by removal of scDNA-seq cells does not provide insights into the behavior of MaCroDNA under extreme conditions where the proportions of clones deviate from the original ones significantly. To address this, we designed another experiment on the CRC patients, in which we randomly drew the clonal proportions in the DNA data and resampled the scDNA-seq cells with replacement within each clone to achieve the drawn proportions. Assuming there are K clones in a CRC patient named P, and their corresponding proportions are denoted by $\Pi_P = [\pi_P^1, \pi_P^2, \cdots, \pi_P^K]$, we performed the following steps:

1. Draw K new proportion for all the DNA clones from the following Dirichlet-multinomial distribution,

$$[\hat{\pi}_P^1, \cdots, \hat{\pi}_P^K] \sim \text{DirMult}(N_P, \boldsymbol{\alpha}_P), \tag{1}$$

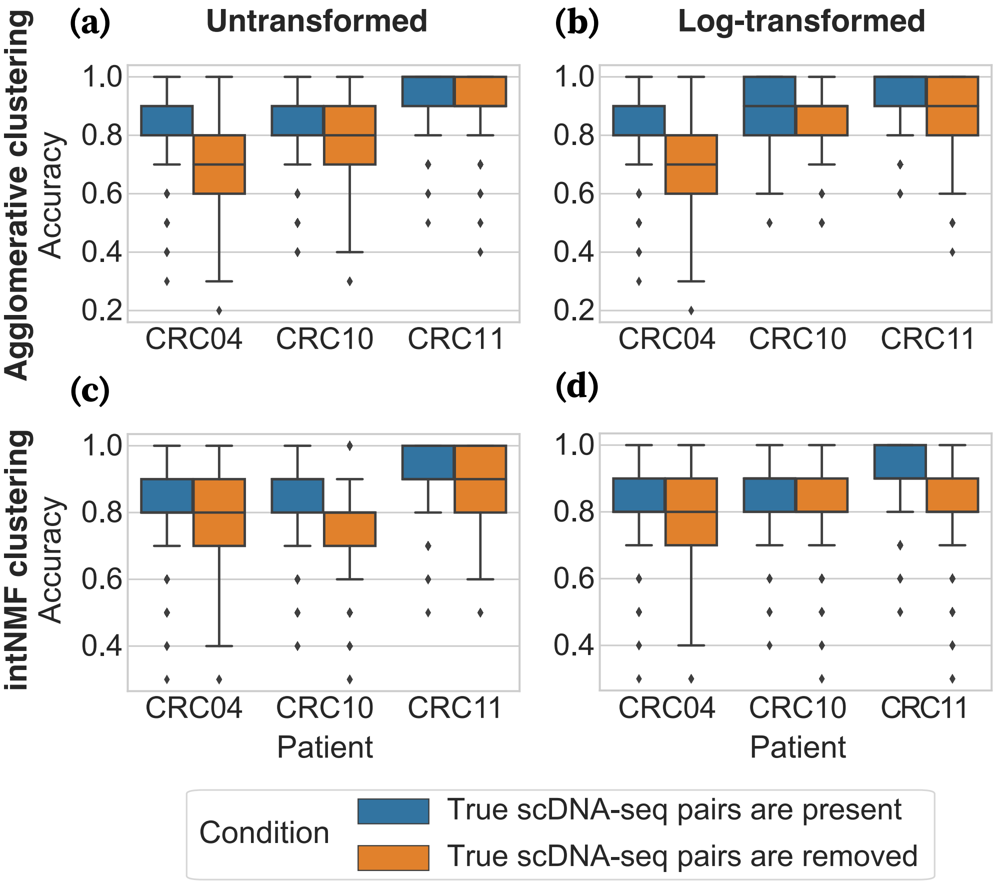

Figure S22: **Accuracy of clonal assignments before and after the random removal of scDNA-seq cells from the DNA data.** For each group of ten randomly selected scDNA-seq cells that we removed from the DNA data, we measured the accuracy of clonal assignments of their true scRNA-seq pairs before and after the removal of those selected scDNA-seq cells. These box plots compare the accuracies under these two conditions for all the patients when the clustering algorithms and their input data were **(a)** agglomerative clustering and the original data, **(b)** agglomerative clustering and log-transformed data, **(c)** intNMF clustering and original data, and **(d)** intNMF clustering and log-transformed data (10,000 random repetitions for each configuration of patient, clustering algorithm, and input data). The results belonging to before and after the removal of scDNA-seq cells are shown in blue and orange boxes, respectively. Each box represents the data points between the 25th and 75th percentiles ($Q1$ and $Q3$, respectively). The whiskers extend to $Q1 - 1.5(Q3 - Q1)$ and $Q3 + 1.5(Q3 - Q1)$. Every data outside of this range is counted as an outlier. Source data are provided as a Source Data file.

where $\hat{\pi}_P^i$ represents the new proportion of clone i in patient P, N_P denotes the total number of scDNA-seq cells in patient P (number of trials for the Dirichlet-multinomial distribution) and $\boldsymbol{\alpha}_P = [\alpha_P^1, \cdots, \alpha_P^K]$ is an all-ones vector of length K containing the parameters of the Dirichlet prior. Here we used a symmetric Dirichlet prior with a concentration parameter of

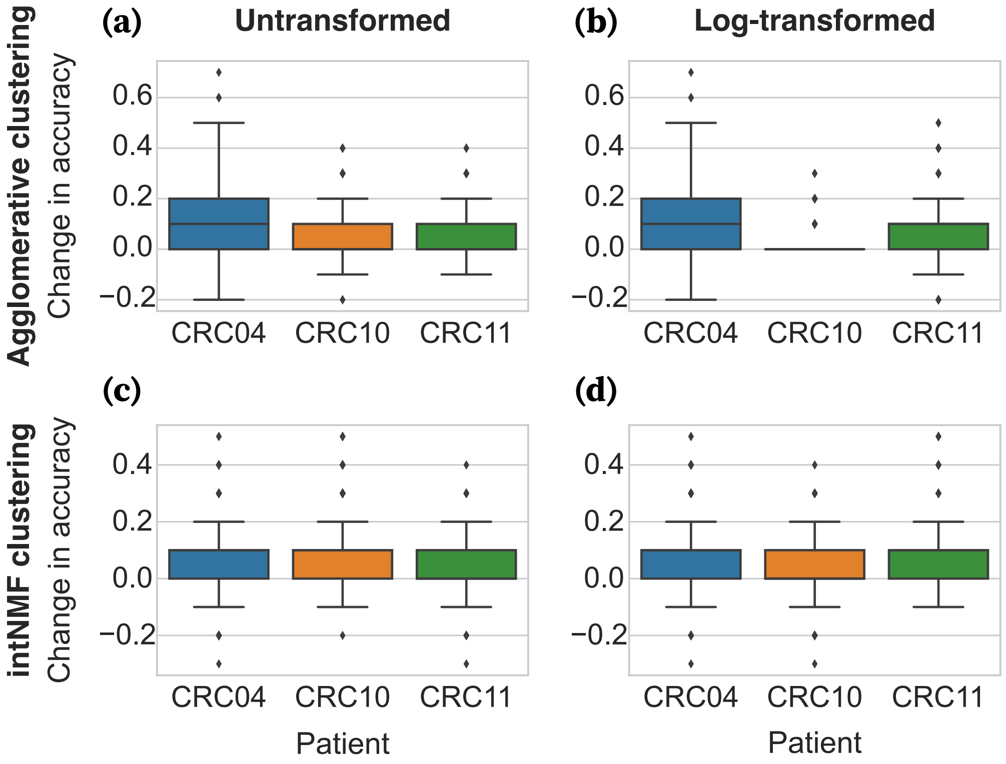

Figure S23: **Effect of the removal of scDNA-seq cells from the DNA input on the clonal assignment accuracy.** For each random removal repetition, we subtracted the clonal assignment accuracy of the ten randomly selected scRNA-seq cells whose true DNA pairs were removed, after the removal, from their clonal assignment accuracy before the removal. Each box plot shows these measures for all the patients when the clustering algorithms and their input data were **(a)** agglomerative clustering and the original data, **(b)** agglomerative clustering and log-transformed data, **(c)** intNMF clustering and original data, and **(d)** intNMF clustering and log-transformed data (10,000 random repetitions for each configuration of patient, clustering algorithm, and input data). The boxes are colored based on their corresponding patient labels. Each box represents the data points between the 25th and 75th percentiles ($Q1$ and $Q3$, respectively). The whiskers extend to $Q1 - 1.5(Q3 - Q1)$ and $Q3 + 1.5(Q3 - Q1)$. Every data outside of this range is counted as an outlier. The negative values show that, in some cases, the removal of scDNA-seq pairs improved the clonal assignment of their RNA pairs. Source data are provided as a Source Data file.

1 ($\boldsymbol{\alpha}_P = \mathbf{1}$).

2. Given the randomly drawn clonal proportions from step 1, for each clone i, sample $\hat{\pi}_P^i$ scDNA-seq cells within that clone using replacement.

3. For patient P, aggregate all randomly sampled scDNA-seq cells and, along with the original RNA data, pass them as input to MaCroDNA.

4. Measure the accuracy of clonal assignments of the scRNA-seq cells with joint RNA-DNA information by counting the number of scRNA-seq cells that are assigned to the same clones that their true scDNA-seq pairs belong to.

5. Repeat steps 1 to 3, 10,000 times.

We performed the above experiment on all patients and all clustering settings with the highest clustering resolution available (four clusters per patient for agglomerative clustering, and the optimal number of clusters for intNMF clustering). Fig. S24 shows the clonal assignment accuracy measurements for all the repetitions, patients, and clustering settings. Although this figure shows that the accuracy of clonal assignments decreases with the randomly drawn clonal proportions, we observed that the variation in the average accuracy was dependent on the choice of clustering technique: while the average accuracy across the tests and patients with agglomerative clustering ranged approximately from 30% to 50% (Fig. S24a and b), the results with intNMF clustering showed higher average accuracy between 60% to 80% (Fig. S24c and d).

To better understand the impact of the difference between the clonal proportions in the two modalities on the clonal assignment accuracy, we calculated the Earth mover's distance (EMD) between the original clonal proportions and the sampled ones for each random test using Python's Scipy package v1.5.2. Next, we calculated the Spearman correlation between the EMDs and the corresponding accuracy values for each patient and clustering setting. Figs. S25, S26, and S27 illustrate the scatter plots of the accuracy measures against the EMD values for patients CRC04, CRC10, and CRC11, respectively. We observed strong negative Spearman correlation coefficients with zero p-values in almost all patients and clustering settings, indicating that the accuracy drops as the clonal proportions deviate from the original ones. The only exception was observed in the data under intNMF clustering in patient CRC04, which demonstrated a moderate negative correlation (see Fig. S25c and d). This is due to the presence of some interesting cases where the accuracy increased with the increase in the EMD.

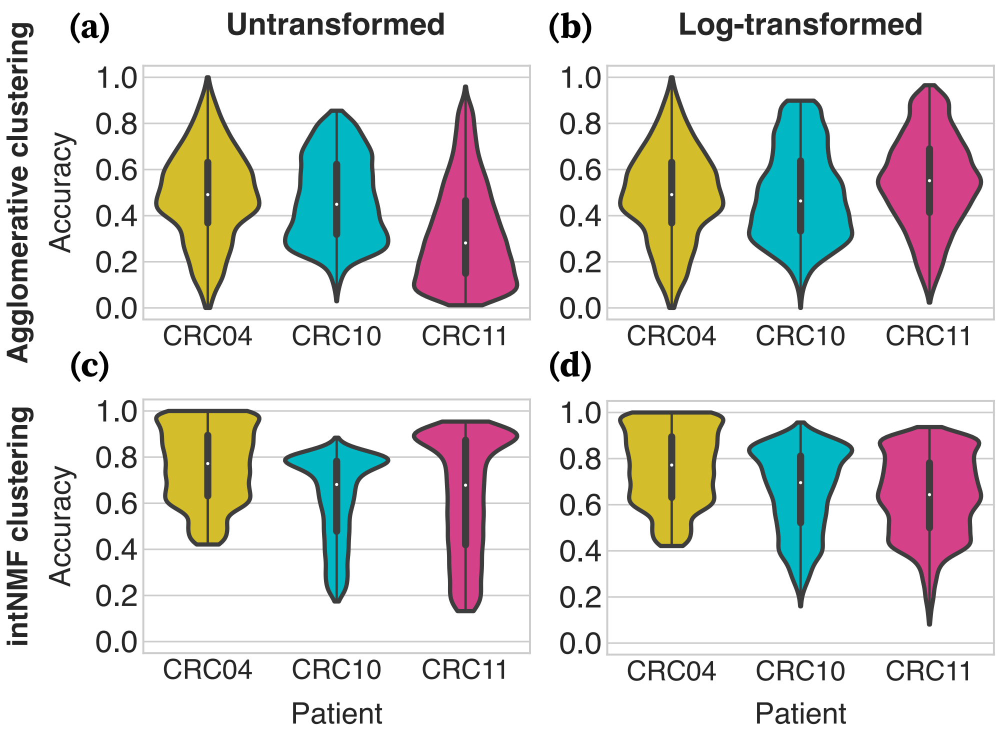

Figure S24: **Accuracy of clonal assignment for different randomly sampled clonal proportions.** Each violin plot shows the accuracy distributions of clonal assignments for scRNA-seq cells with both RNA and DNA information in each patient when the clustering algorithms and their input data were **(a)** agglomerative clustering and the original data, **(b)** agglomerative clustering and log-transformed data, **(c)** intNMF clustering and original data, and **(d)** intNMF clustering and log-transformed data (10,000 random repetitions for each configuration of patient, clustering algorithm, and input data). The violins are colored based on their patient labels. On each violin, the white dot represents the median. The thick black bar in the center of the violin shows the interquartile range (data between the 25th and 75th percentiles, $Q1$ and $Q3$, respectively. The black lines stretched from the interquartile bar extend to $Q1 - 1.5(Q3 - Q1)$ and $Q3 + 1.5(Q3 - Q1)$, minimum and maximum, respectively. Source data are provided as a Source Data file.

3.2 Random assignment test for BE biopsies

Since the ground truth information of the paired scDNA-seq and scRNA-seq cells was not provided for the BE biopsies, and we could not evaluate the correctness of the scRNA-seq cells' assignments, obtaining the confidence scores for the scRNA-seq cells' assignments was necessary and useful for the analysis. As a simple way of determining the confidence scores, for each biopsy, we compared the result of MaCroDNA with those of a large number of random assignments replicates. To randomly assign scRNA-seq cells to scDNA-seq ones, we used the same rules for the maximum

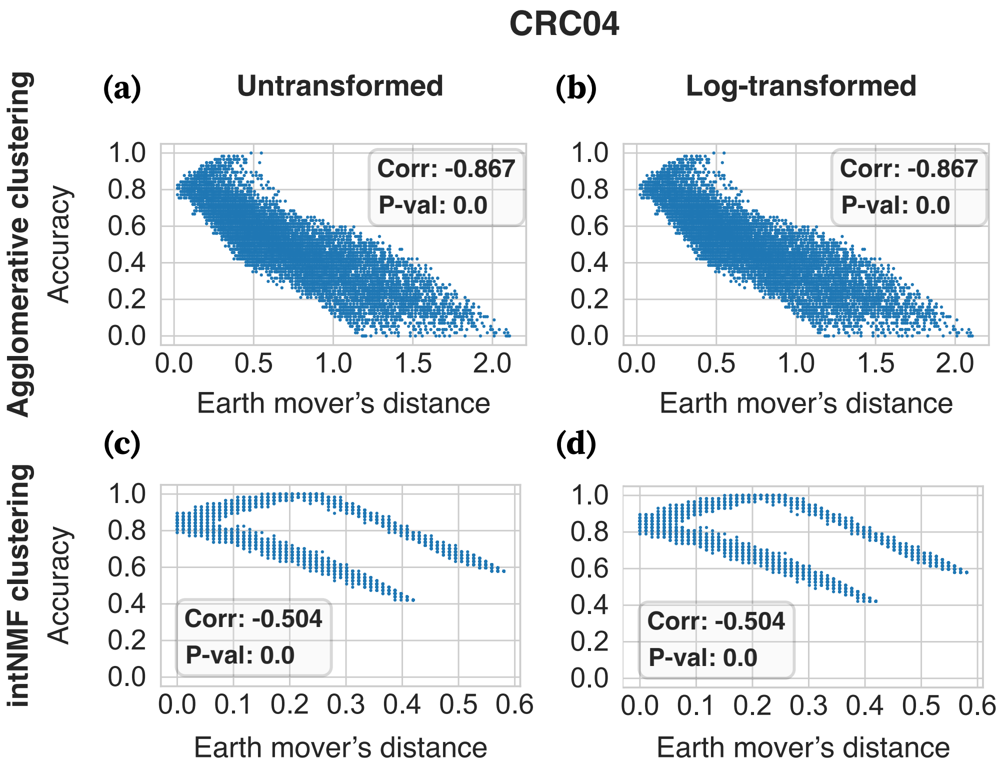

Figure S25: **Correlation between the clonal assignment accuracy and EMD of the randomly sampled and the original clonal proportions in patient CRC04**. Each dot in the scatter plots illustrates the clonal assignment accuracy against the EMD of the sampled and original clonal proportions in a single random sampling. The four panels correspond to the clustering algorithms and their input data: **(a)** agglomerative clustering and the original data, **(b)** agglomerative clustering and log-transformed data, **(c)** intNMF clustering and original data, and **(d)** intNMF clustering and log-transformed data. We performed 10,000 random repetitions for each configuration of patient, clustering algorithm, and input data ($n = 93$ DNA cells in CRC04 patient). In each panel, the Spearman correlation coefficient and its p-value are shown in the corner of the panel. The p-values are estimated by a two-sided hypothesis test whose null hypothesis is that accuracy and Earth mover's distance have no correlation. The null distribution was a Student t-distribution with $\nu = 9,998$ degree of freedom for 10,000 random repetitions. Under the agglomerative clustering setting, we observed a strong negative correlation indicating that the accuracy significantly drops as the clonal proportions deviate from the original ones. Although the data under the intNMF clustering displayed an overall decreasing trend, they demonstrated a moderate negative correlation, due to the presence of some cases where the accuracy increases with the EMD. Source data are provided as a Source Data file.

number of scRNA-seq cells assigned to each scDNA-seq cell: if $N_{\mathbf{G}}$ and $N_{\mathbf{C}}$ denote the number of scRNA-seq and scDNA-seq cells, respectively, then each scDNA-seq cell is allowed to be selected by at most $\lceil \frac{N_{\mathbf{G}}}{N_{\mathbf{C}}} \rceil$ scRNA-seq cells. Following this rule, we repeated this random assignment for each

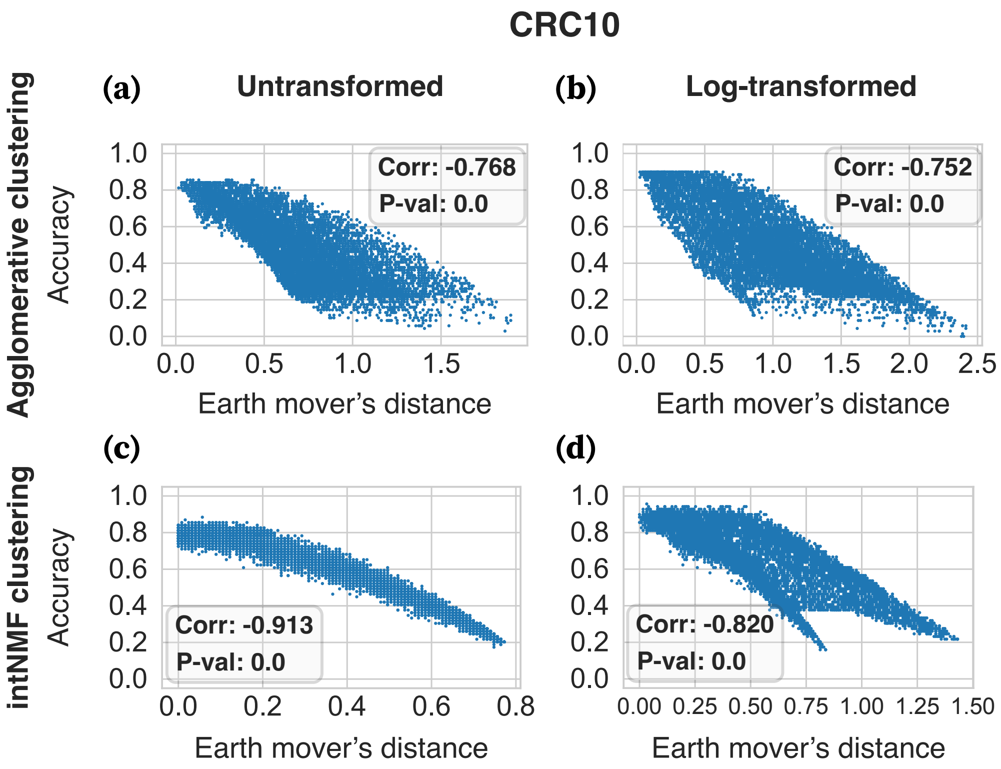

Figure S26: **Correlation between the clonal assignment accuracy and EMD of the randomly sampled and the original clonal proportions in patient CRC10.** Each dot in the scatter plots illustrates the clonal assignment accuracy against the EMD of the sampled and original clonal proportions in a single random sampling. The four panels correspond to the clustering algorithms and their input data: **(a)** agglomerative clustering and the original data, **(b)** agglomerative clustering and log-transformed data, **(c)** intNMF clustering and original data, and **(d)** intNMF clustering and log-transformed data. We performed 10,000 random repetitions for each configuration of patient, clustering algorithm, and input data ($n = 123$ DNA cells in CRC10 patient). In each panel, the Spearman correlation coefficient and its p-value are shown in the corner of the panel. The p-values are estimated by a two-sided hypothesis test whose null hypothesis is that accuracy and Earth mover's distance have no correlation. The null distribution was a Student t-distribution with $\nu = 9,998$ degree of freedom for 10,000 random repetitions. In the patient CRC10, we observed a strong negative correlation indicating that the accuracy significantly drops as the clonal proportions deviate from the original ones. Source data are provided as a Source Data file.

biopsy, 100 million times. For both the random assignments and MaCroDNA, we used the sum of the Pearson correlation coefficients between the assigned cells as the score of the assignment. Next, for each biopsy, we calculated the p-value of MaCroDNA's assignments as the proportion of random assignment scores that were greater than MaCroDNA's score, divided by the total number

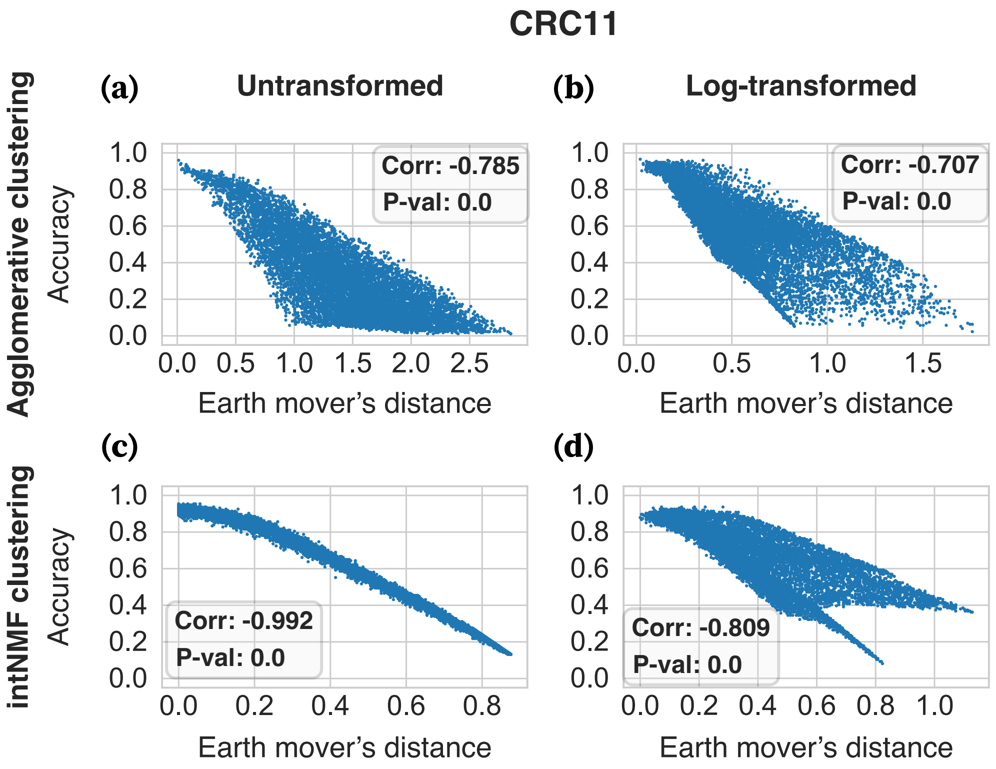

Figure S27: **Correlation between the clonal assignment accuracy and EMD of the randomly sampled and the original clonal proportions in patient CRC11.** Each dot in the scatter plots illustrates the clonal assignment accuracy against the EMD of the sampled and original clonal proportions in a single random sampling. The four panels correspond to the clustering algorithms and their input data: **(a)** agglomerative clustering and the original data, **(b)** agglomerative clustering and log-transformed data, **(c)** intNMF clustering and original data, and **(d)** intNMF clustering and log-transformed data. We performed 10,000 random repetitions for each configuration of patient, clustering algorithm, and input data ($n = 249$ DNA cells in CRC11 patient). In each panel, the Spearman correlation coefficient and its p-value are shown in the corner of the panel. The p-values are estimated by a two-sided hypothesis test whose null hypothesis is that accuracy and Earth mover's distance have no correlation. The null distribution was a Student t-distribution with $\nu = 9,998$ degree of freedom for 10,000 random repetitions. Similar to patient CRC10, in patient CRC11, we observed a strong negative correlation indicating that the accuracy significantly drops as the clonal proportions deviate from the original ones. Source data are provided as a Source Data file.

of random assignment replicates. The histograms of the scores for all BE biopsies, along with the calculated p-values of MaCroDNA's scores, are shown in Fig. S28. As shown, all p-values for MaCroDNA's scores are zero, indicating the non-triviality of the MaCroDNA's results compared to the random assignments.

Since the sum of Pearson correlation coefficients might be affected by the outlier values, we further used the median of Pearson correlation values among the paired cells as the score of an assignment (for both MaCroDNA and random assignments). Fig. S29 illustrates the histograms of medians for all the BE biopsies. We used the same random seed to reproduce the random assignments that are shown in Fig. S28. Similar to the previous experiment, the p-value for the MaCroDNA median was calculated as the proportion of random assignments' medians that were greater than MaCroDNA's median, divided by the total number of random assignment replicates. As illustrated, the median of Pearson correlation coefficients inferred by MaCroDNA is still higher than those of the random assignments and the zero p-values in all BE biopsies imply that MaCroDNA's results are statistically significant.

The visual inspection of Figs. S28 and S29 suggested that using median instead of sum, did not change the ranking of BE biopsies in terms of their random assignment scores, i.e., the healthy and non-dysplastic BE biopsies had relatively lower scores than the higher-degree biopsies, based on both the sum and the median of Pearson correlation values. To quantify this observation, we drew the regression plot between the two scores and calculated the Spearman and Pearson correlation values between them. Specifically, for each BE biopsy, we measured the median of random assignments' scores for each of the two metrics including the median and sum of Pearson correlation values, as the summary scores of that BE biopsy. Fig. S30 shows that the median of randomly drawn scores (sum and median) are highly correlated with significant p-values (the correlation values and their corresponding p-values are shown at the top of the figure). This implies that the original distributions of Pearson correlation values in the random assignments were not skewed.

More importantly, Fig. S30 shows that, overall, random assignment of cells on healthy and non-dysplastic BE biopsies obtained lower correlation values compared to the high-degree and cancer biopsies. This implies that two randomly drawn cells, one with gene expression data and the other with copy number information from a healthy or non-dysplastic biopsy, tend to demonstrate less correlation compared to two randomly drawn cells from high-degree or cancer biopsies. Although the level of heterogeneity in these biopsies seems to be a contributing factor (we discuss the effect

of heterogeneity on the performance of MaCroDNA in Sections Stability analysis of MaCroDNA's assignments for BE biopsies and Retrieval of BE biopsy labels), here, heterogeneity does not affect a random assignment scheme as this scheme does not prioritize the assignment of cells with higher correlation. To have a higher correlation in random assignments, there must be copy number changes shared by a large portion of DNA cells that drive differential gene expressions (such CNAs might have occurred at the early stages of clonal expansion and disease development). Under this scenario, random assignments show high correlations even if the biopsy is homogeneous. Therefore, we speculate that the presence of (or lack thereof) gene-expression-influencing copy number changes present in major DNA clones might be the key factor (see clone-specific CNAs in high-degree and cancer biopsies in Figures 2 and 3 of the original study [2]). Of course, our hypothesis requires more investigation and we leave it as future direction.

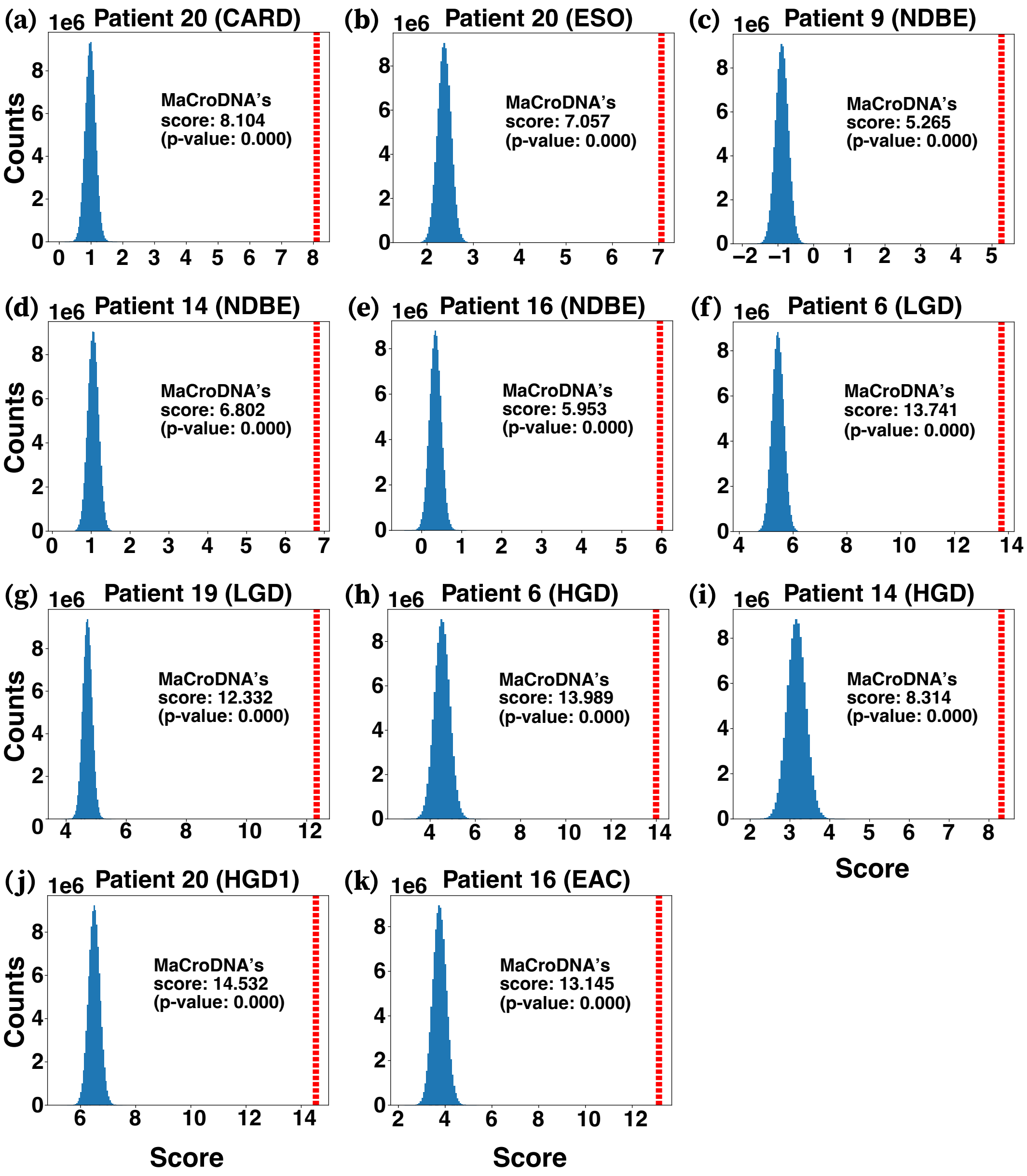

Figure S28: **Histograms of random assignment scores for the BE biopsies.** Each panel illustrates the histogram of the 100 million random assignment scores for a BE biopsy: **(a-b)** for healthy biopsies, 20 (CARD) and 20 (ESO); **(c-e)** for non-dysplastic biopsies, 9 (NDBE), 14 (NDBE), and 16 (NDBE); **(f-g)** for low-grade biopsies, 6 (LGD) and 19 (LGD); **(h-j)** for high-grade biopsies, 6 (HGD), 14 (HGD), and 20 (HGD1); and **(k)** for cancer biopsy 16 (EAC). The x-axis represents the scores, and the y-axis represents the corresponding counts. In each panel, a red vertical line is plotted to MaCroDNA's score. Additionally, the p-value of MaCroDNA's score is displayed on each biopsy panel. For each biopsy, the p-value of MaCroDNA's assignments was estimated by a one-sided permutation test as the proportion of random assignment scores that were greater than MaCroDNA's score, divided by the total number of random assignment replicates (sample sizes were the total number of DNA and RNA cells per biopsy: $n = 591$ for 20 (CARD), $n = 520$ for 20 (ESO), $n = 522$ for 9 (NDBE), $n = 459$ for 14 (NDBE), $n = 464$ for 16 (NDBE), $n = 574$ for 6 (LGD), $n = 671$ for 19 (LGD), $n = 501$ for 6 (HGD), $n = 289$ for 14 (HGD), $n = 572$ for 20 (HGD), and $n = 461$ for 20 (EAC)). The presence of a large gap between the random and MaCroDNA's scores, along with all p-values being zero, indicates that MaCroDNA's assignments are non-trivial. Source data are provided as a Source Data file.

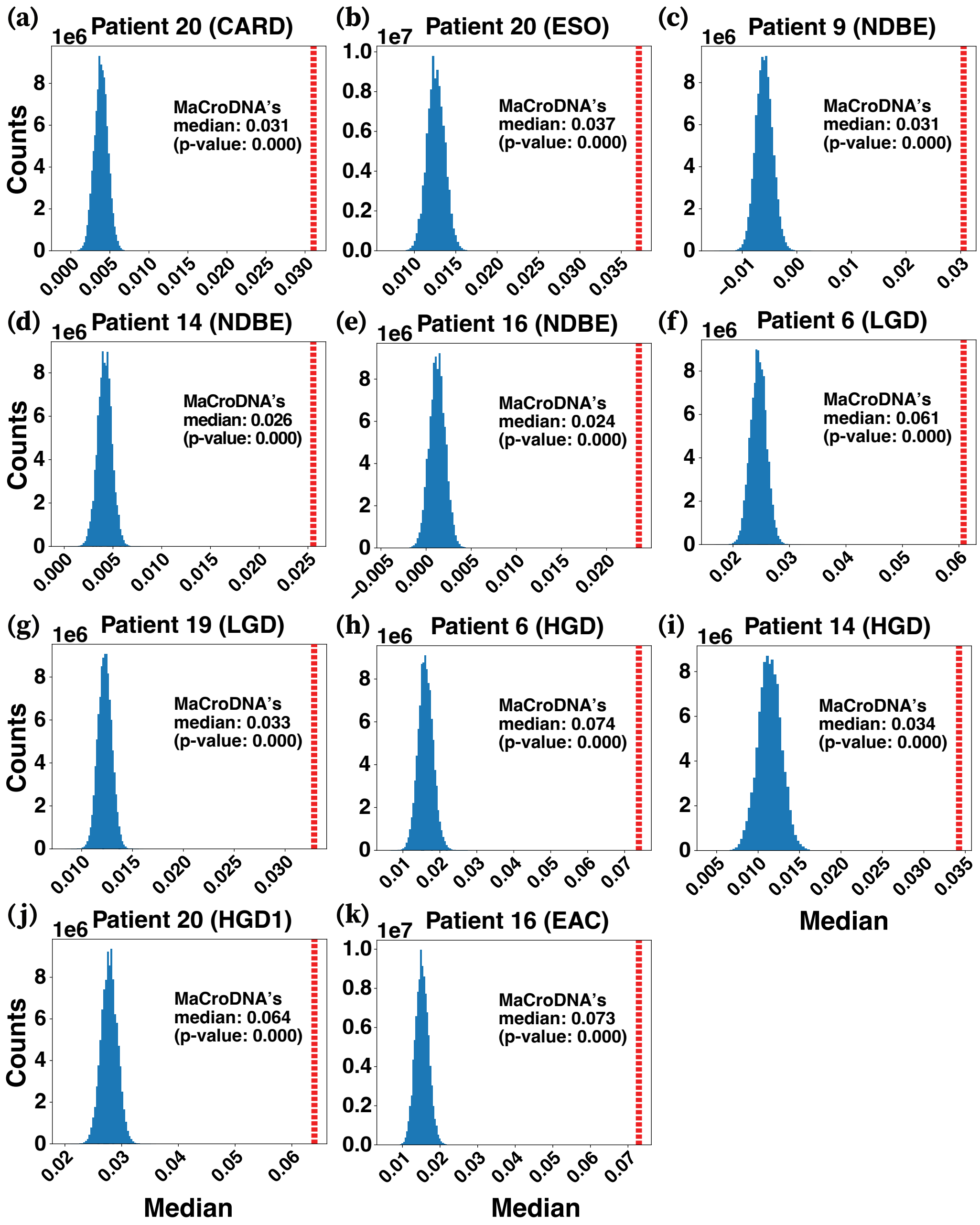

Figure S29: **Histograms of median of Pearson correlation coefficients among all paired cells for each random assignment in each BE biopsy.** Each panel illustrates the histogram of the medians for the 100 million random assignments performed for each BE biopsy (the same trials as in Fig. S28): **(a-b)** for healthy biopsies, 20 (CARD) and 20 (ESO); **(c-e)** for non-dysplastic biopsies, 9 (NDBE), 14 (NDBE), and 16 (NDBE); **(f-g)** for low-grade biopsies, 6 (LGD) and 19 (LGD); **(h-j)** for high-grade biopsies, 6 (HGD), 14 (HGD), and 20 (HGD1); and **(k)** for cancer biopsy 16 (EAC). The x-axis represents the median values, and the y-axis represents the corresponding counts. In each panel, a red vertical line is plotted to show MaCroDNA's median. Additionally, the p-value of MaCroDNA's median is displayed on each biopsy panel. For each biopsy, the p-value of MaCroDNA's assignments was estimated by a one-sided permutation test as the proportion of random assignment scores that were greater than MaCroDNA's score, divided by the total number of random assignment replicates (sample sizes were the same as in Fig. S28). The presence of a large gap between the random and MaCroDNA's medians, along with all p-values being zero, indicates that MaCroDNA's assignments are non-trivial. Source data are provided as a Source Data file.

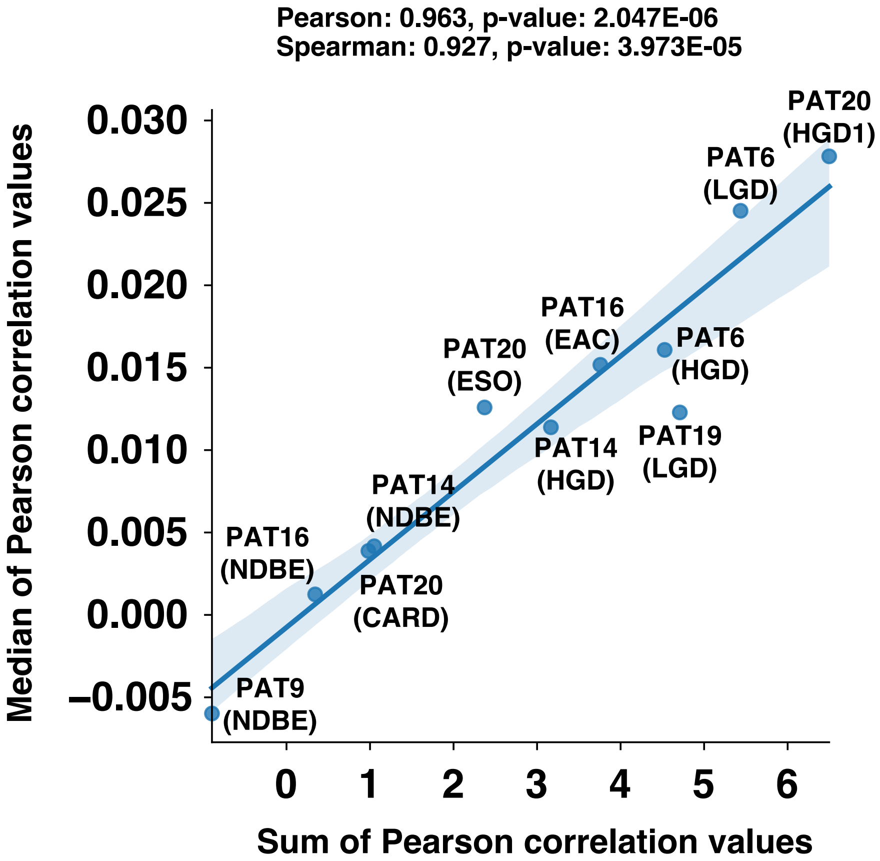

Figure S30: **Regression plot between the median of random assignments' scores (the sum and median of Pearson correlation values) for all BE biopsies.** Each dot represents a BE biopsy. For each biopsy, we measured the median of random assignments' scores for the two metrics including the median of Pearson correlation values (y-axis), and sum of Pearson correlation values (x-axis). The regression line is in blue, and the 95% confidence interval is shown in light blue shaded area. The Pearson and Spearman correlation coefficients, along with their p-values are shown at the top of this figure. For both Pearson and Spearman correlations, the p-values are estimated by a two-sided hypothesis test whose null hypothesis is that two scores have no correlation ($n = 11$ patients). The null distribution was a Student t-distribution with $\nu = 9$ degree of freedom for eleven patients. The strong and significant correlation between the two scores indicates that the original distributions of Pearson correlation values in the random assignments were not skewed. Moreover, one can observe that the healthy and non-dysplastic biopsies demonstrate lower scores compared to the higher-degree biopsies according to both scores. Source data are provided as a Source Data file.

3.3 Stability analysis of MaCroDNA's assignments for BE biopsies

The BE data set contains biopsies from healthy, diseased, and cancer tissues. It is reasonable to assume that as the level of heterogeneity in the scDNA-seq data of a biopsy decreases, and the copy number profiles become more identical, selecting the best match for each scRNA-seq cell in the RNA data becomes less definitive. To study this hypothesis, we defined two measures; one for the stability and definitiveness of the scRNA-seq assignments named *assignment instability index* (AII), and the other for measuring the level of heterogeneity of a biopsy, based on the pairwise L1-norm distances between its scDNA-seq cells' copy number profiles. Finally, we investigated the correlation between the two measures. In the following sections, first, we describe the AII and our comparison between the biopsies in terms of this index, and next, the definition of the heterogeneity score and its relationship with the AII.

3.3.1 Assignment instability index

To measure the stability of scRNA-seq cells' assignments in the BE biopsies, we designed a leave-one-out experiment that involved introducing a small perturbation into the input data by leaving out one of the scRNA-seq cells. Specifically, for each scRNA-seq cell g_i, in a biopsy, we performed the following:

1. Remove g_i from the RNA input. Run MaCroDNA on the rest of the RNA data and the entire DNA data in the biopsy.

2. Assign g_i to the scDNA-seq cell to which less than $\lceil \frac{N_{\mathbf{G}}}{N_{\mathbf{C}}} \rceil$ scRNA-seq cells are already assigned, and it has the highest Pearson correlation coefficient with g_i. Here, $N_{\mathbf{G}}$ and $N_{\mathbf{C}}$ denote the total number of scRNA-seq and scDNA-seq cells in the biopsy, respectively.

Such perturbation in the RNA inputs might change the assignment of not only the left-out scRNA-seq cell but also the remaining scRNA-seq cells. Therefore, for each scRNA-seq cell, we aggregated the scDNA-seq cells' IDs assigned to it across all the leave-one-out trials. Next, for each scRNA-seq cell, we collected the scDNA-seq cell IDs that were different from the assigned scDNA-seq cell ID when running MaCroDNA on the entire RNA data and defined the AII as the number of such

different scDNA-seq cell IDs. The higher the AII, the more unstable and indefinitive the assignment of a scRNA-seq is in the presence of perturbation. As illustrated in Fig. S31, the distribution of assignment instability indices across all biopsies is almost identical. However, they differ in the outliers.

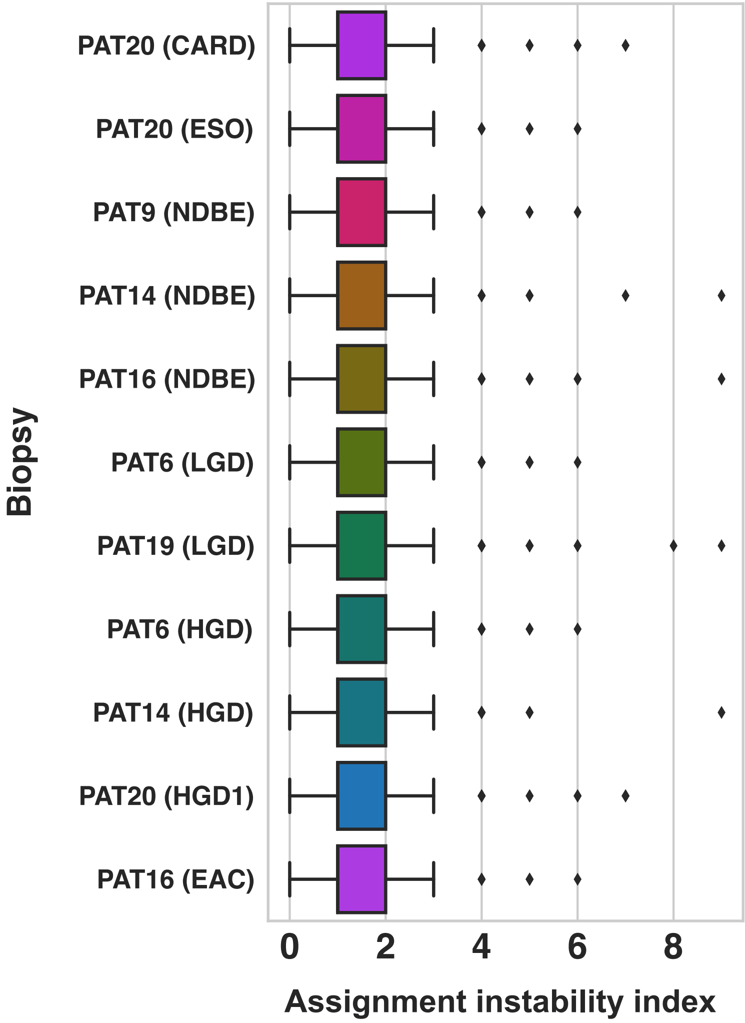

Figure S31: **Box plots of the assignment instability indices for the BE biopsies.** The biopsy labels are shown on the y-axis, and the x-axis shows the assignment instability index values observed for all the scRNA-seq cells in the biopsy across all leave-one-out trials. The distributions are almost identical except for the outliers. Each box represents the data points between the 25th and 75th percentiles ($Q1$ and $Q3$, respectively). The whiskers extend to $Q1 - 1.5(Q3 - Q1)$ and $Q3 + 1.5(Q3 - Q1)$. Every data outside of this range is counted as an outlier. Source data are provided as a Source Data file.

3.3.2 Maximum assignment instability indices are highly correlated with heterogeneity scores

To investigate the potential relationship between the stability of the assignments with the level of copy number heterogeneity in the BE biopsies, we needed to define a heterogeneity measurement. First, for each biopsy, we calculated the pairwise L1-norm distances between all scDNA-seq cells; then, to avoid the effect of outliers, instead of the mean, we used the median of the distribution of the pairwise distances as the heterogeneity score. As mentioned in the previous section, we hypothesized that the outliers might be the differentiating features between the distributions of AIIs across the BE biopsies. Therefore, we summarized each biopsy's AII distribution with its range of values (difference between maximum and minimum values). Since the minimum AII in all biopsies was zero, the maximum AII became the representative of the biopsies' AII values. Calculating the Pearson and Spearman correlation coefficients showed a strong negative correlation between the maximum AIIs and the heterogeneity scores, suggesting that more similarity between the copy number profiles makes the assignment of scRNA-seq cells more indefinitive and conversely, more heterogeneous biopsies demonstrate more stable and definitive assignments (Fig. S32).

3.4 Retrieval of BE biopsy labels

As demonstrated in the previous experiment, the level of heterogeneity in the BE biopsies affects the definitiveness of the assignments. Having this observation, we sought to investigate the definitiveness of the assignments, this time, across the biopsies by aggregating all the scDNA-seq and scRNA-seq cells from all biopsies and running MaCroDNA on the pooled data without providing the cells' biopsy labels to the method. This way, we allowed the scRNA-seq cells to be assigned to any biopsy's scDNA-seq cell. Having the true biopsy labels, we measured the accuracy of assignments per biopsy, as the number of the scRNA-seq cells that are assigned to a scDNA-seq cell from the same biopsy divided by the total number of scRNA-seq cells in the biopsy. Table S10 shows the accuracy of each biopsy in this experiment. Among the BE biopsies, all healthy and non-dysplastic biopsies displayed very low accuracy while one of the low-grade, two of the high-grade, and the cancer biopsies demonstrated high accuracy values (see the green rows of Table. S10). This

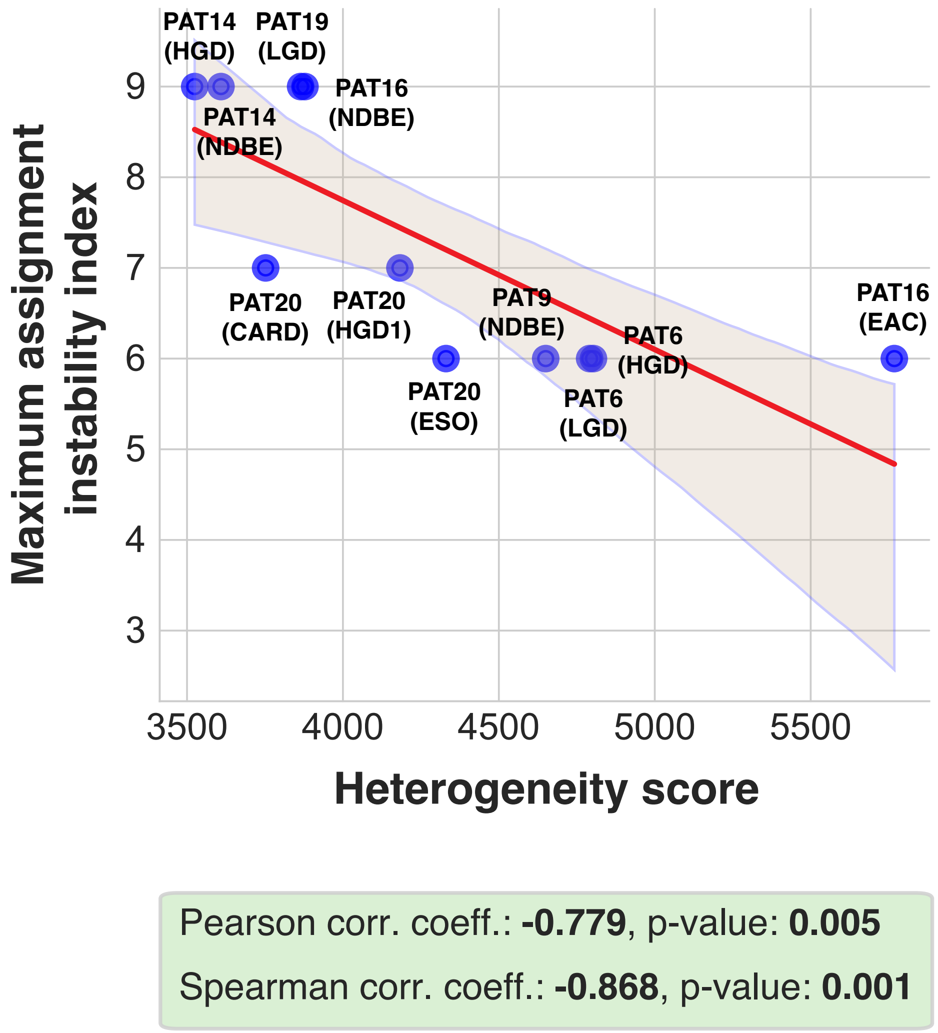

Figure S32: **Regression plot between the maximum assignment instability indices and heterogeneity scores of the BE biopsies.** Each dot represents a biopsy. The heterogeneity scores and assignment instability indices are shown on the x-axis and y-axis, respectively. The regression line is in red, and the 95% confidence interval is shown by the brown shaded area. The Pearson and Spearman correlation coefficients, along with their p-values, are shown in the green box at the bottom of this figure. For both Pearson and Spearman correlations, the p-values are estimated by a two-sided hypothesis test whose null hypothesis is that two scores have no correlation ($n = 11$ patients). The null distribution was a Student t-distribution with $\nu = 9$ degree of freedom for eleven patients. The correlation coefficients imply that lower heterogeneity in a biopsy makes the scRNA-seq assignments more indefinitive. Source data are provided as a Source Data file.

clearly suggests that the definitiveness and confidence of the assignments increases with the level of heterogeneity in the biopsies.

Additionally, we looked into the distribution of each biopsy's scRNA-seq cells' assignments to

Biopsy	Accuracy (%)
PAT20 (CARD)	12.85
PAT20 (ESO)	26.51
PAT9 (NDBE)	13.12
PAT14 (NDBE)	10.42
PAT16 (NDBE)	23.20
PAT6 (LGD)	77.37
PAT19 (LGD)	14.68
PAT6 (HGD)	76.19
PAT14 (HGD)	27.90
PAT20 (HGD1)	88.41
PAT16 (EAC)	80.21

Table S10: **Accuracy of biopsies' label retrieval.** The high accuracy values are observed in one of the low-grade (PAT6 (LGD)), two of the high-grade (PAT6 (HGD) and PAT20 (HGD1)), and the cancer (PAT16 (EAC)) biopsies, while all healthy and non-dysplastic biopsies displayed very low accuracy. Source data are provided as a Source Data file.

the same biopsy and the others. As shown in Fig. S33, starting from the healthy and non-dysplastic biopsies, the distribution of assignment counts to all biopsies deviate from uniformity and become more concentrated around the same biopsy; this means more scRNA-seq cells were assigned to the same biopsies as the biopsies become more heterogeneous. Moreover, we observed the same relationship between heterogeneity and the assignment accuracy of the biopsies; the biopsies with less accuracy displayed a more uniform distribution, and inversely, the biopsies with more accuracy had a distribution more concentrated around their own label.

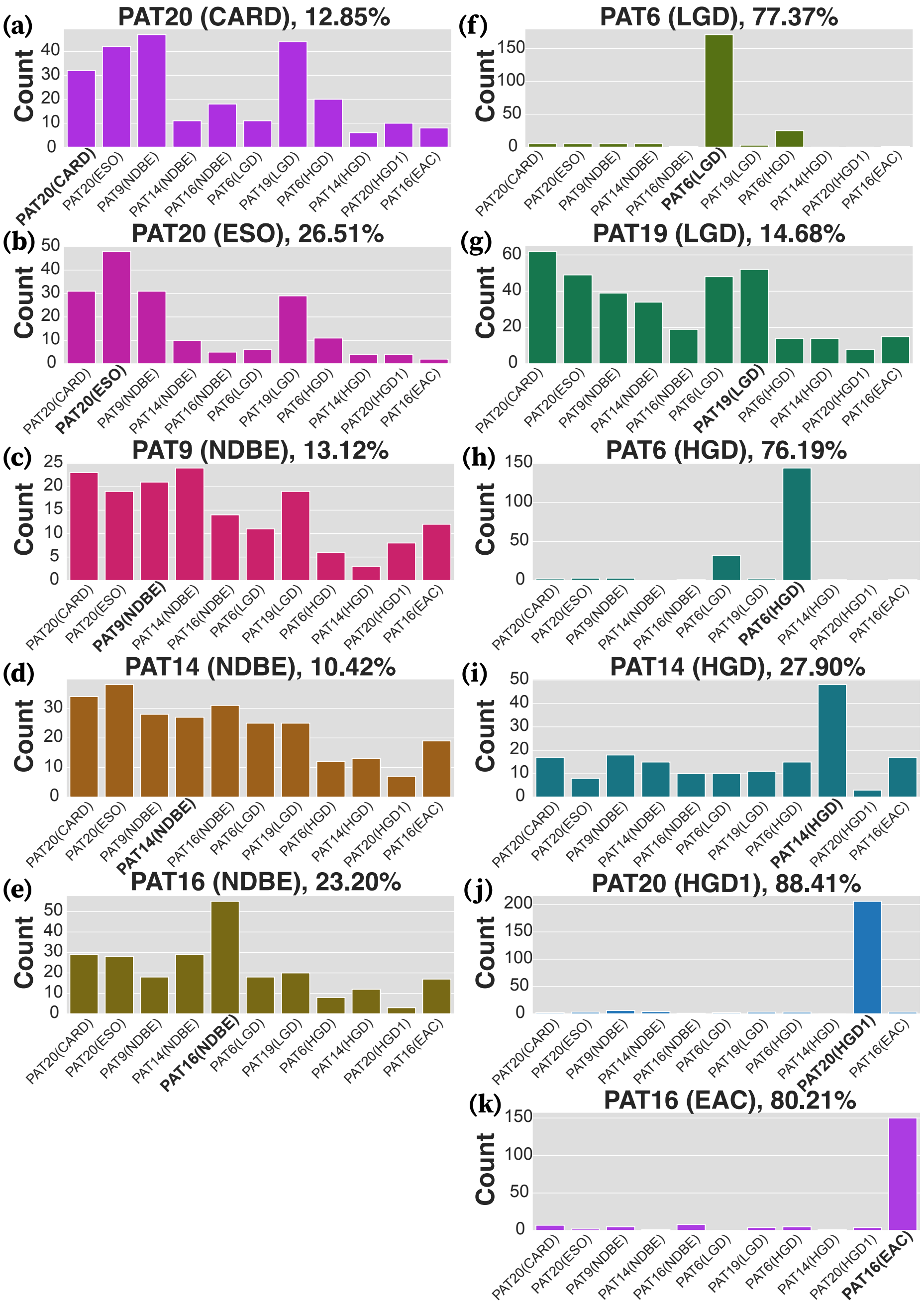

Figure S33: **Distribution of scRNA-seq cells' assignments to all biopsies.** Each panel corresponds to a BE biopsy and illustrates the number of the biopsy's scRNA-seq cells that were assigned to scDNA-seq cells from the same or the other biopsies. The biopsy s assignment accuracy is shown on top of each panel. The y-axis shows the number of assigned scRNA-seq cells, and the x-axis shows the biopsy labels of the scDNA-seq cells that the scRNA-seq cells were assigned to. Moving from the healthy and non-dysplastic biopsies **(a-e)** to low-grade, high-grade, and cancer biopsies **(f-k)**, the distributions deviate from uniformity and become more concentrated around the same biopsy implying that MaCroDNA could assign more scRNA-seq cells to the same biopsy. Source data are provided as a Source Data file.

Supplementary References

[1] Simon P Blomberg, Theodore Garland Jr, and Anthony R Ives. Testing for phylogenetic signal in comparative data: behavioral traits are more labile. *Evolution*, 57(4):717–745, 2003.

[2] Georg A Busslinger, Buys de Barbanson, Rurika Oka, Bas LA Weusten, Michiel de Maat, Richard van Hillegersberg, Lodewijk AA Brosens, Ruben van Boxtel, Alexander van Oudenaarden, and Hans Clevers. Molecular characterization of Barrett's esophagus at single-cell resolution. *Proceedings of the National Academy of Sciences*, 118(47):e2113061118, 2021.
