# Supplementary material for: Accurate integration of single-cell DNA and RNA for analyzing intratumor heterogeneity using MaCroDNA
Source: Nat Commun. 2023 Dec 13;14:8262. doi: 10.1038/s41467-023-44014-3 (PMC10719311; doi:10.1038/s41467-023-44014-3)
Supplement: Supplementary file 3 — Reporting Summary [file 41467_2023_44014_MOESM3_ESM.pdf]

nature portfolio

Corresponding author(s): Mohammadamin Edrisi, Huw A. Ogilvie, Luay Nakhleh

Last updated by author(s): 11/16/2023

Reporting Summary

Nature Portfolio wishes to improve the reproducibility of the work that we publish. This form provides structure for consistency and transparency in reporting. For further information on Nature Portfolio policies, see our Editorial Policies and the Editorial Policy Checklist.

Statistics

For all statistical analyses, confirm that the following items are present in the figure legend, table legend, main text, or Methods section.

n/a	Confirmed	
☐	☒	The exact sample size (*n*) for each experimental group/condition, given as a discrete number and unit of measurement
☐	☒	A statement on whether measurements were taken from distinct samples or whether the same sample was measured repeatedly
☐	☒	The statistical test(s) used AND whether they are one- or two-sided<br>*Only common tests should be described solely by name; describe more complex techniques in the Methods section.*
☒	☐	A description of all covariates tested
☒	☐	A description of any assumptions or corrections, such as tests of normality and adjustment for multiple comparisons
☐	☒	A full description of the statistical parameters including central tendency (e.g. means) or other basic estimates (e.g. regression coefficient) AND variation (e.g. standard deviation) or associated estimates of uncertainty (e.g. confidence intervals)
☐	☒	For null hypothesis testing, the test statistic (e.g. *F*, *t*, *r*) with confidence intervals, effect sizes, degrees of freedom and *P* value noted<br>*Give P values as exact values whenever suitable.*
☒	☐	For Bayesian analysis, information on the choice of priors and Markov chain Monte Carlo settings
☒	☐	For hierarchical and complex designs, identification of the appropriate level for tests and full reporting of outcomes
☐	☒	Estimates of effect sizes (e.g. Cohen's *d*, Pearson's *r*), indicating how they were calculated

Our web collection on statistics for biologists contains articles on many of the points above.

Software and code

Policy information about availability of computer code

Data collection	No software was used for data collection.
Data analysis	Our new open-source MaCroDNA software (v1.0.0) was used for integration of single-cell DNA-seq and single-cell RNA-seq data sets after copy number/gene expression quantification. The MaCroDNA v1.0.0 package is publicly available on GitHub (https://github.com/NakhlehLab/MaCroDNA) and is archived on Zenodo (https://zenodo.org/doi/10.5281/zenodo.10115041).<br>The scripts underlying our analyses are written in either Python v3.8.5 or R v4.2.2<br><br>Gurobipy package (v 10.0.0, https://pypi.org/project/gurobipy/) was used for optimization via Integer Linear Programming in Python.<br><br>Software and Python packages used for clustering analysis on the colorectal cancer (CRC) copy number data from Bian et al. (2018):<br>intNMF v1.2.0 (https://cran.r-project.org/web/packages/IntNMF/index.html) was used for clustering based on non-negative matrix factorization.<br>Scipy v1.5.2 (https://scipy.org) and scikit-learn v0.23.2 (https://pypi.org/project/scikit-learn/) were used for agglomerative hierarchical clustering in Python.<br><br>Packages used for data visualization:<br>Matplotlib v3.3.2 (https://matplotlib.org/stable/) and seaborn v0.11.0 (https://seaborn.pydata.org) in Python.<br>ggplot2 R package v 3.4.1 (https://cran.r-project.org/web/packages/ggplot2/index.html).<br><br>We used phylosignal R package v1.3 (https://cran.r-project.org/web/packages/phylosignal/index.html) to quantify phylogenetic signal.<br>The following libraries were imported for phylosignal package as dependencies:

adephylo v1.1.13 (https://cran.r-project.org/web/packages/adephylo/index.html).
ape v5.7 (https://cran.r-project.org/web/packages/ape/index.html).
phylobase v0.8.10 (https://cran.r-project.org/web/packages/phylobase/index.html).

phangorn R package v2.11.1 (https://cran.r-project.org/web/packages/phangorn/index.html) was used for inference of phylogenetic tree (UPGMA) for Barrett's esophagus copy number data from the original study by Busslinger et al. (2021).

Software used for the integration of CRC copy number and gene expression from Bian et al. (2018) :
clonealign package v0.99.0 (https://github.com/kieranrcampbell/clonealign)
Seurat v4.3.0 (https://cran.r-project.org/src/contrib/Archive/Seurat/)
CCNMF package (https://github.com/XQBai/CCNMF). No version number is provided on the GitHub repository of CCNMF.

The other Python packages include:
Numpy v1.19.2 (https://numpy.org) for numerical computing.
Pandas v1.1.3 (https://pandas.pydata.org) for dataframe manipulation.
Scanpy v1.7.1 (https://scanpy.readthedocs.io/en/stable/index.html) for preprocessing on gene expression data.

Software used in the original study by Bian et al. (2018) for read mapping and copy number/gene expression quantification:
For the single-cell DNA methylome sequencing data,
Bismark v0.20.0 (https://github.com/FelixKrueger/Bismark) for sequence alignment of BS-seq reads of single-cell DNA methylome sequencing data.
Samtools v1.9 (https://sourceforge.net/projects/samtools/files/samtools/) for sorting and removing the duplicates.
Bedtools v2.27.1 (https://bedtools.readthedocs.io/en/latest/) for converting the BAM files to BED files.
Ginkgo (https://github.com/robertaboukhalil/ginkgo) to infer the absolute copy number values. No version number is provided on the GitHub repository of Ginkgo.
Tophat v2.1.1 (http://ccb.jhu.edu/software/tophat/index.shtml) was used for mapping RNA reads.

Software used in the original study by Busslinger et al. (2021) for read mapping and copy number/gene expression quantification:
The NlaIII mapping pipeline of SingleCellMultiOmics package (v0.1.22): https://github.com/BuysDB/SingleCellMultiOmics/tree/master/singlecellmultiomics/snakemake_workflows/nlaIII was used for mapping the single-cell DNA sequencing reads.
The reads of the scRNA-seq data were mapped to the human genome using the SingleCellMultiOmics pipeline (v0.1.22): https://github.com/BuysDB/SingleCellMultiOmics/tree/master/singlecellmultiomics/snakemake_workflows/cs2_scmo

For manuscripts utilizing custom algorithms or software that are central to the research but not yet described in published literature, software must be made available to editors and reviewers. We strongly encourage code deposition in a community repository (e.g. GitHub). See the Nature Portfolio guidelines for submitting code & software for further information.

Data

Policy information about availability of data

All manuscripts must include a data availability statement. This statement should provide the following information, where applicable:

- Accession codes, unique identifiers, or web links for publicly available datasets
- A description of any restrictions on data availability
- For clinical datasets or third party data, please ensure that the statement adheres to our policy

The CRC data from Bian et al. (2018) is openly available in NCBI Gene Expression Omnibus (GEO) under accession number GSE97693 (https://www.ncbi.nlm.nih.gov/geo/query/acc.cgi?acc=GSE97693). The BE data set from Busslinger et al. (2021) is available in European Genome-Phenome Archive (EGA) under accession number EGAS00001005221 (https://ega-archive.org/studies/EGAS00001005221). Access to this data is controlled by a Data Access Committee.
RNA and DNA read counts for both the CRC and BE were obtained directly from the authors of the original studies.
The GENCODE GFF3 annotation file for GRCh37 assembly was downloaded from https://www.gencodegenes.org/human/release_19.html.
The list of cancer-related genes used in this study were downloaded from the COSMIC Cancer Gene Census web page at https://cancer.sanger.ac.uk/census.
The data associated with the figures presented in this study are provided in the Source Data file. Source data are provided with this paper.

Research involving human participants, their data, or biological material

Policy information about studies with human participants or human data. See also policy information about sex, gender (identity/presentation), and sexual orientation and race, ethnicity and racism.

Reporting on sex and gender	No new human data was collected for our study so this does not apply.
Reporting on race, ethnicity, or other socially relevant groupings	No new human data was collected for our study so this does not apply.
Population characteristics	No new human data was collected for our study so this does not apply.
Recruitment	No new human data was collected for our study so this does not apply.
Ethics oversight	No new human data was collected for our study so this does not apply.

Note that full information on the approval of the study protocol must also be provided in the manuscript.

Field-specific reporting

Please select the one below that is the best fit for your research. If you are not sure, read the appropriate sections before making your selection.

☒ Life sciences ☐ Behavioural & social sciences ☐ Ecological, evolutionary & environmental sciences

For a reference copy of the document with all sections, see nature.com/documents/nr-reporting-summary-flat.pdf

Life sciences study design

All studies must disclose on these points even when the disclosure is negative.

Sample size	No statistical methods were used to predetermine the sample sizes. The sample sizes were the patient tumors/biopsies sequenced for both RNA and DNA, and the number of cells sequenced in those biopsies, from the original CRC study by Bian et al. and BE study by Busslinger et al. after data exclusions (below) which resulted in n=3 patients' data sets from the CRC study by Brian at el., and n=11 biopsies from the BE study of Busslinger et al. We did not choose any specific sample sizes but rather aimed to collect as many samples with ground truth information as possible to serve as a reliable benchmark data for which CRC data set was the only published study (containing 370 scRNA-seq cells and 465 scDNA-seq cells). The BE data set contains 2442 scRNA-seq cells and 3182 scDNA-seq cells from six patients with different stages of Barrett's esophagus disease which makes one of the largest and most diverse data sets in the literature.
Data exclusions	As we did not collect any original data, we did not pre-establish exclusion protocols. Two CRC tumors were excluded due to use of a different sequencing protocol, and one was excluded due to an insufficient number of sequenced RNA cells. Cells in the BE data set with fewer than 3,000 reads were excluded as having insufficient data to map across omics domains.
Replication	We ran our method on the CRC data for evaluation under 64 different conditions by changing the clustering techniques (agglomerative clustering and intNMF), preprocessing on clustering methods' inputs, gene selection techniques, and clustering resolutions for agglomerative clustering. We confirm that under all these conditions, our method performed better than the existing methods.<br>For the resampling experiments, including random removal of scDNA-seq cells and resampling clonal proportions for CRC data, and random assignment tests for BE biopsies, we made the results reproducible by fixing the random seeds in all codes.
Randomization	Each method was applied to exactly the same set of CRC tumors so randomization was not necessary. Visualization and qualitative analysis of phylogenetic signal in BE biopsies was a post-hoc analysis rather than experimental.
Blinding	Blinding was not performed as only n=3 CRC tumors were used to compare method accuracy, and only n=1 to n=3 BE biopsies were available for each grade, so we restricted our analysis to visualization and qualitative interpretation. In the absence of original data collection or formal statistical testing of that data, blinding was unnecessary.

Reporting for specific materials, systems and methods

We require information from authors about some types of materials, experimental systems and methods used in many studies. Here, indicate whether each material, system or method listed is relevant to your study. If you are not sure if a list item applies to your research, read the appropriate section before selecting a response.

Materials & experimental systems

n/a	Involved in the study	
☒	☐	Antibodies
☒	☐	Eukaryotic cell lines
☒	☐	Palaeontology and archaeology
☒	☐	Animals and other organisms
☒	☐	Clinical data
☒	☐	Dual use research of concern
☒	☐	Plants

Methods

n/a	Involved in the study	
☒	☐	ChIP-seq
☒	☐	Flow cytometry
☒	☐	MRI-based neuroimaging

Plants

Seed stocks	No plant material was used or collected for our study so this does not apply.
Novel plant genotypes	No plant material was used or collected for our study so this does not apply.
Authentication	No plant material was used or collected for our study so this does not apply.
